# Bibliometric analysis of neutrophil extracellular traps induced by protozoan and helminth parasites (2008–2024)

**DOI:** 10.3389/fimmu.2025.1498453

**Published:** 2025-01-24

**Authors:** Tamara Muñoz-Caro, Elvira M. Saraiva, Rafael M. Mariante

**Affiliations:** ^1^ Escuela de Medicina Veterinaria, Facultad de Medicina Veterinaria y Recursos Naturales, Universidad Santo Tomás, Talca, Chile; ^2^ Laboratório de Imunidade Inata, Instituto de Microbiologia Paulo de Góes, Universidade Federal do Rio de Janeiro, Rio de Janeiro, Brazil; ^3^ Laboratório de Biologia Estrutural, Instituto Oswaldo Cruz, Fiocruz, Rio de Janeiro, Brazil

**Keywords:** NETs, neutrophils, protozoa, heminths, bibliometrics, VOSviewer, Biblioshiny, CiteSpace

## Abstract

**Introduction:**

Parasitic diseases pose a significant global public health challenge, affecting billions of people and causing substantial economic losses in livestock and poultry. In the fight against these infections, neutrophils play a crucial role, employing various strategies, including the release of neutrophil extracellular traps (NETs). Recent studies have made significant progress in understanding NETs triggered by protozoa and helminths. However, a comprehensive bibliometric analysis that compiles these findings and identifies research hotspots and trends in this field is still lacking.

**Methods:**

We utilized the Web of Science Core Collection and Scopus databases to retrieve original articles on NETs induced by protozoa and helminths. After screening, the data was transferred to the visualization tools VOSviewer, Biblioshiny, and CiteSpace for analysis.

**Results:**

Our study included 159 original articles published in 69 journals, involving 909 authors from 270 institutions across 41 countries. Germany and Brazil have made the most significant contributions to the research on NETs and parasites, accounting for 45 and 32 publications, and 1,495 and 1,342 citations, respectively. Carlos Hermosilla and Anja Taubert (Justus Liebig University Giessen, Germany), and Elvira Saraiva (Federal University of Rio de Janeiro, Brazil), are leaders in the field, both in terms of publication output and citations. *Frontiers in Immunology* has consistently and significantly impacted the field, and an article in the *Proceedings of the National Academy of Sciences of the United States of America* providing the first direct evidence of NETs release in response to a parasite is by far the most cited. Current research focuses on malaria, *Toxoplasma gondii*, *Besnoitia besnoiti*, nematodes, and the mechanisms of NETs production and their effects on parasites and host cells. Emerging trends include therapeutic targeting of NETs and comparative studies across different host and parasite species.

**Conclusion:**

This study offers a comprehensive overview and visual analysis of NETs and parasites, highlighting key areas for future research.

## 1 Introduction

In 2004, a seminal work published by Volker Brinkmann et al. from the Max Planck Institute for Infection Biology in Germany described a new biological phenomenon involving the release by neutrophils of a structure composed of chromatin and associated proteins, capable of trapping and killing bacteria. This structure was named neutrophil extracellular traps, or NETs (1). This paper has received impressive recognition, being cited almost 7,000 times. Since its publication, various research groups have been dedicated to understanding the mechanisms involved in the process of NETs release and investigating which other stimuli can induce the formation of such structures. With just over two decades of studies and thousands of articles published on the subject, we now know that the production of NETs occurs in response to microorganisms of all groups, including various species of bacteria, fungi, viruses, protozoa, and helminths. Nevertheless, although it constitutes a crucial defense mechanism for maintaining the host organism free from pathogens, the excessive production of NETs can trigger or worsen various diseases, including systemic lupus erythematosus, diabetes, preeclampsia, atherosclerosis, thrombosis, cancer, and COVID-19, to name a few (2).

Infections caused by parasites, including protozoa and helminths, constitute a serious public health problem, affecting billions of people worldwide (3, 4). Additionally, they cause significant economic losses, leading to malnutrition, weight loss, reproductive issues, and sometimes death in livestock and poultry (5, 6). In response to infections, neutrophils migrate to the infection site and combat microbes using various strategies, including phagocytosis, generation of reactive oxygen species (ROS), release of antimicrobial content from their granules, production of cytokines, attraction of other immune cells, and the release of NETs (7). The mechanisms driving neutrophil responses in the context of parasitic infections are not yet fully understood. In protozoan infections, several studies have shown the involvement of neutrophil recruitment to control parasite dissemination. However, exacerbation of the neutrophil response can lead to host tissue damage and enhance disease pathogenesis (8). In helminth infections, studies on neutrophil responses are even more limited. Although several studies have shown neutrophil recruitment and activity in the early stages of infection, their contribution to the subsequent immune response, including crosstalk with other immune cells, and tissue damage requires further investigation (9). In both cases, a growing body of evidence indicates an important role for NETs release during infections.

Bibliometric analysis is a powerful research tool that quantitatively evaluates the impact and trends within a specific field by analyzing publications, citations, and collaborative networks. It offers valuable insights into research productivity, identifies influential contributors and institutions, and uncovers emerging themes in the literature (10). Combining mathematical and statistical methods with data from scientific databases allows for clear visualization of various aspects of research within a given field. In this study, we conducted a bibliometric analysis of the research on NETs in protozoan and helminth infections from 2008 to 2024. Our objectives were to identify the most prolific authors, countries, institutions, and journals, explore research hotspots and development trends, and ultimately provide researchers with new ideas and collaboration opportunities.

## 2 Methods

### 2.1 Data acquisition

This bibliometric analysis was conducted in accordance with the guidelines outlined in previous studies (11, 12). All raw data used in this work was publicly available, and there was no need for review by an ethics committee. The data were obtained from the Web of Science Core Collection and Scopus, widely used academic databases known for their rigorous curation and selection standards (12). We searched for all papers related to NETs and protozoan or helminth parasites.

To ascertain the quality of the data retrieved, we used a variety of topic keywords and Boolean operators in the Topic (TS) field: (“extracellular trap*” OR “extracellular DNA trap*” OR etosis OR netosis) AND (parasite* OR protozoa* OR apicomplexa* OR coccid* OR trypanosom* OR trichomon* OR diplomonad* OR malaria OR toxoplasm* OR cryptosporid* OR leishman* OR giard* OR amoeb* OR ameb* OR entamoeb* OR entameb* OR acanthamoeb* OR acanthameb* OR plasmodium OR besnoitia OR eimeria OR neospora OR naegleria OR helminth* OR fluke* OR nematod* OR trematod* OR cestod* OR worm* OR roundworm* OR hookworm* OR whipworm* OR tapeworm* OR larva* OR ascaris OR trichuris OR ancylostoma OR necator OR mesocestoides OR echinococcus OR strongyl* OR nippostrongyl* OR toxocara OR haemonchus OR trichinella OR brugia OR ostertagia OR filaria* OR dirofilaria* OR litomosoides OR onchocerca OR schistosoma OR fasciola OR opisthorchis). Note that keyword variations are retrieved by using an asterisk (*) after a keyword. For example, “helminth*” will retrieve “helminth”, “helminths”, and “helminthiasis”, in addition to other variations. Exclusion criteria included studies on extracellular traps other than those produced by neutrophils and studies focusing on parasites that infect invertebrate hosts.

**Figure 1:**
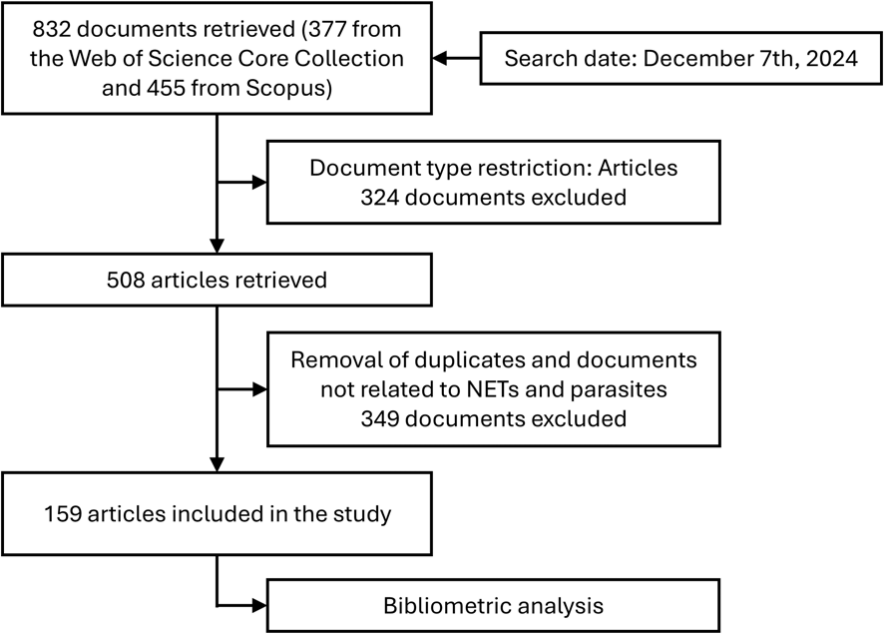
Flowchart of the screening process.

The search was conducted on December 7, 2024, without any time or language restrictions. It retrieved 377 documents from the Web of Science and 455 documents from Scopus. A filter was applied to include only “Articles” in the document types, resulting in 254 documents from each database. The files were exported to Rayyan, a web-based application that facilitates collaborative literature reviews (https://www.rayyan.ai/). After removing duplicates and individually excluding works unrelated to the defined subject, a total of 159 articles were retained. Subsequently, we returned to the Web of Science and Scopus to manually select the papers filtered with Rayyan. The cleaned files from both databases were extracted and combined using the *bibliometrix* package in RStudio, as previously described (12). The combined file was then analyzed as outlined below. An overview of the data collection and analysis process is presented in **Figure 1**.

### 2.2 Data analysis

In this study, we used VOSviewer, Biblioshiny, and CiteSpace to compute and graph the data obtained from the Web of Science Core Collection and Scopus. VOSviewer, developed for constructing and viewing bibliometric maps (13), was used here (version 1.6.20) to create and analyze network maps of countries, institutions, journals, authors, and keywords. Bibliometrix is an open-source tool built in R designed for performing comprehensive science mapping analysis (14). We used its web interface, Biblioshiny, run in R (version 4.3.3), to visually analyze the countries’ collaboration research world map, the keywords, the trend topics, and the thematic map in the field of NETs and parasites. *Thesaurus* and *synonym* files were created and imported into VOSviewer or Biblioshiny, respectively, to unify spelling variations and synonyms. CiteSpace is a tool written in Java for visualizing and analyzing the potential knowledge contained in the scientific literature (15). We used CiteSpace (version 6.3.R1 Basic) to analyze literature bursts over time. Finally, we used Microsoft Excel (version 2407) and GraphPad Prism (version 10.1.2) to tabulate the data and create additional graphs.

## 3 Results

### 3.1 Publications and citations over the years

**Figure 2:**
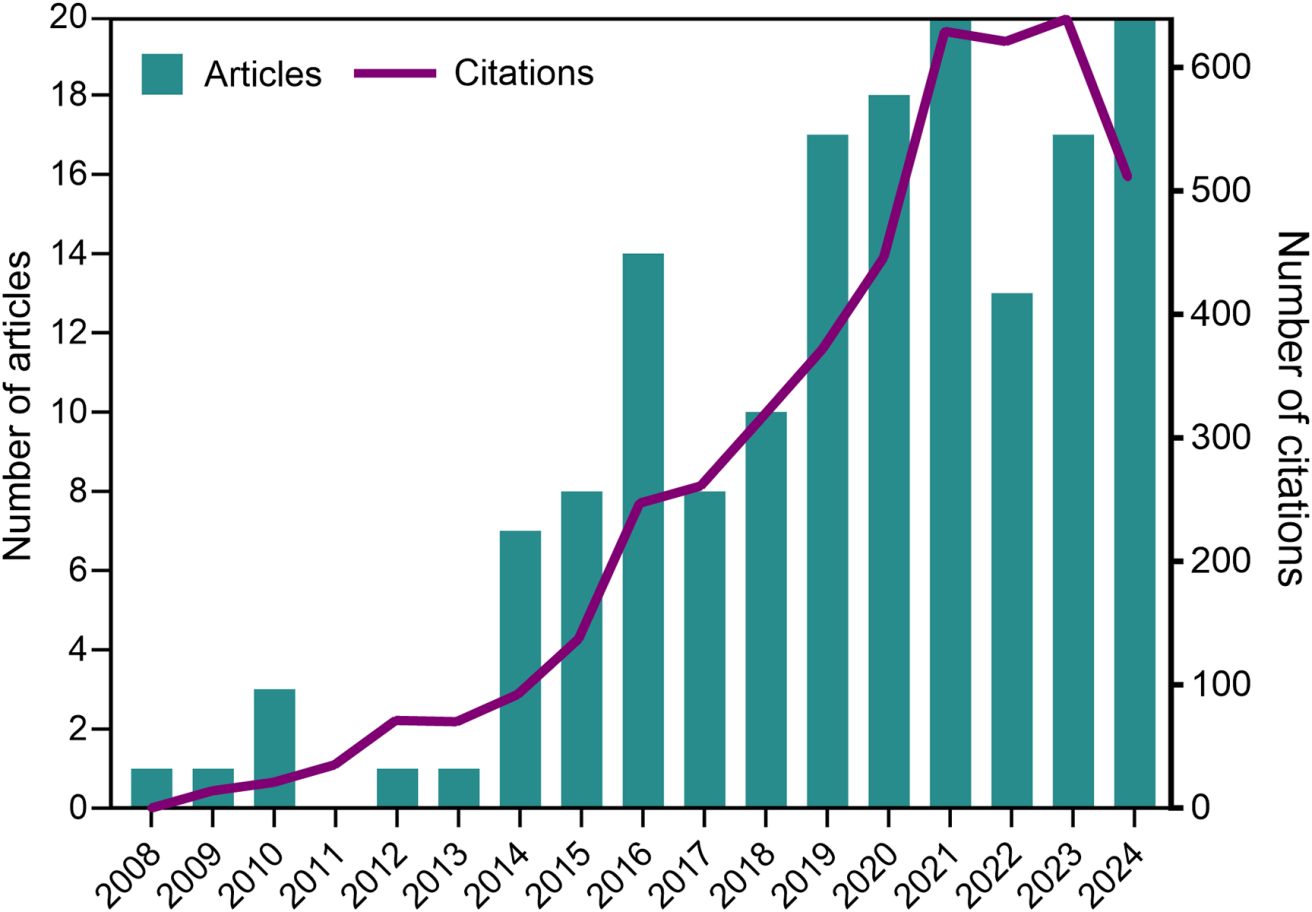
Number of original articles and citations on NETs and protozoan or helminth parasites for each year from 2008 to 2024 (partial data for 2024). Data source: Web of Science Core Collection and Scopus.

Following the flowchart depicted in **Figure 1**, we retrieved 159 articles on this research subject, covering the period from 2008, when the first paper on NETs and parasites was published, to 2024. Overall, we observed an increasing trend in annual publications over the analyzed period. The maximum number of publications in a single year occurred in both 2021 and 2024, with 20 papers each year. The annual citation count generally followed the trend in annual publications, with a slight decline in 2024 (which includes only partial data for December and is expected to increase by the end of the year). The highest number of citations was observed in 2023, with a total of 639 citations. **Figure 2** shows the number of articles and citations on NETs and parasites for each year from 2008 to 2024.

### 3.2 Distribution of countries, institutions and journals

#### 3.2.1 Countries

**Figure 3:**
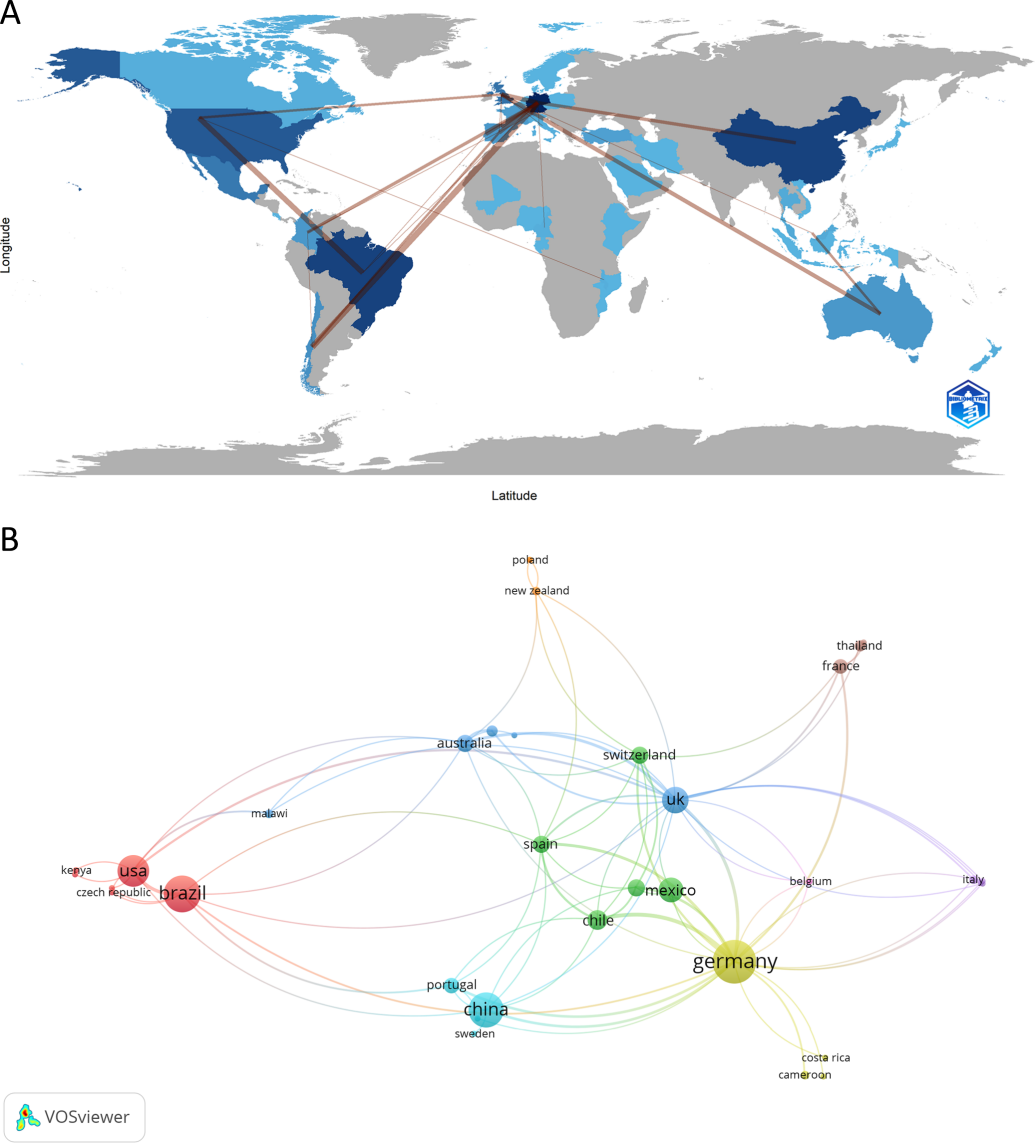
Countries contribution to NETs and parasite studies. **(A)** Countries involved in this area are highlighted in blue, with darker blue representing the most prolific countries. Lines between countries represent their connections. **(B)** Collaborative network map of countries involved in the research subject. The color of the nodes represents countries within the same cluster, while the size of the nodes represents the number of documents. The thickness of the lines corresponds to the strength of inter-country collaboration.

Initially, we investigated the contributions of different countries to NETs and parasite studies. Among the 41 countries with publications in this research field, Germany stands out as the most productive, with the highest number of articles published (n = 45, corresponding to 28.30% of the total articles) and also the highest number of total citations (n = 1,495). **Figure 3A** shows the countries involved in NETs and parasites-related research and their collaborations. In the country network shown in **Figure 3B**, the size of the nodes indicates the number of documents. Connections between nodes indicate collaboration among researchers from different countries, with the thickness of the lines representing the strength of the collaboration. A close collaboration between authors from Germany and Chile can be observed. In terms of the number of published articles, Brazil and China rank next (n = 32, 20.13%), followed by the USA (n = 24, 15.09%). Brazil is also second in the ranking for the number of citations (n = 1,342), followed by the USA (n = 1,147), with which it has closer collaboration. **Table 1** and **Supplementary Table 1** show the top 10 countries in this research area ranked by the number of publications or citations, respectively. **Supplementary Figure 1** shows the average publication year and the average number of citations for each country in the network.

**Table 1:** Top 10 countries contributing to the research area ranked by the number of articles published followed by the number of citations.

Rank	Country	Articles	Citations	Citations/Articles
1	Germany	45	1,495	33,22
2	Brazil	32	1,342	41,94
3	China	32	337	10,53
4	USA	24	1,147	47,79
5	UK	17	713	41,94
6	Mexico	15	293	19,53
7	Chile	9	185	20,56
8	Australia	7	280	40,00
9	Spain	7	198	28,29
10	Switzerland	7	197	28,14

#### 3.2.2 Institutions

**Figure 4:**
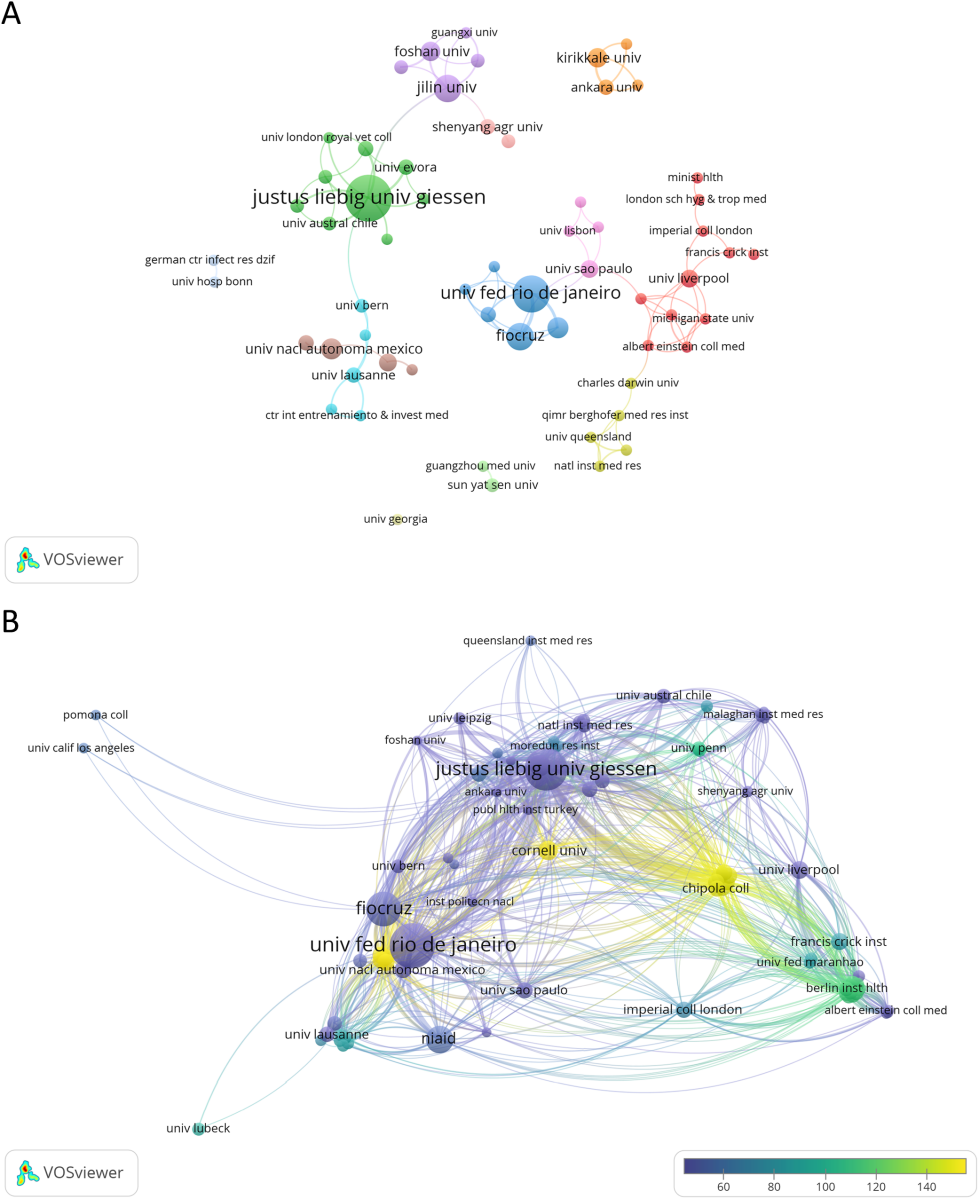
Contribution of research institutions to NETs and parasite studies. **(A)** Collaborative network map of institutions that have published at least two articles in this area. Node colors represent organizations within the same cluster, while node sizes represent the number of documents. **(B)** Collaborative network map of institutions whose documents have garnered 50 or more citations. Node colors reflect the average citations per document published by each institution, while their sizes indicate the institution’s total citation count. Line thickness corresponds to the strength of inter-institution collaboration.

A total of 270 institutions conducted research on NETs and parasites, with 27 (10.00%) of them producing three or more documents on the subject. Justus Liebig University Giessen in Germany leads in terms of articles produced (n = 35, 21.01%), followed by the Federal University of Rio de Janeiro (n = 22, 13.84%) and Fiocruz (n = 12, 7.55%) in Brazil, and Jilin University (n = 12, 7.55%) in China. Among the most cited institutions, the Federal University of Rio de Janeiro holds the first position (n = 1,105 citations), followed by Justus Liebig University Giessen (n = 995), and Fiocruz (n = 636). The principal collaborations among institutions include the Federal University of Rio de Janeiro and Fiocruz (link strength of 9), the Federal University of Rio de Janeiro and the National Institute of Allergy and Infectious Diseases in the USA (link strength of 6), and the following pairs, each with a link strength of 4: Justus Liebig University Giessen and University Santo Tomás in Chile, Justus Liebig University Giessen and University of Évora in Portugal, and Ankara University and Kırıkkale University, both in Turkey. **Figure 4** and **Supplementary Figure 2** show the network of organizations involved in NETs and parasite-related research and their collaborations. **Table 2** and **Supplementary Table 2** list the top 10 most productive institutions contributing to this research field ranked by the number of publications or citations, respectively.

**Table 2:** Top 10 most productive institutions contributing to the research field ranked by the number of articles followed by the number of citations.

Rank	Institution	Country	Articles	Citations
1	Justus Liebig University Giessen	Germany	35	995
2	Federal University of Rio de Janeiro	Brazil	22	1105
3	Fiocruz	Brazil	12	636
4	Jilin University	China	12	235
5	National Institute of Allergy and Infectious Diseases	USA	7	412
6	National Autonomous University of Mexico	Mexico	7	157
7	Kırıkkale University	Turkey	7	58
8	Foshan University	China	6	56
9	University of Liverpool	UK	5	174
10	University of São Paulo	Brazil	5	142

#### 3.2.3 Journals

**Figure 5:**
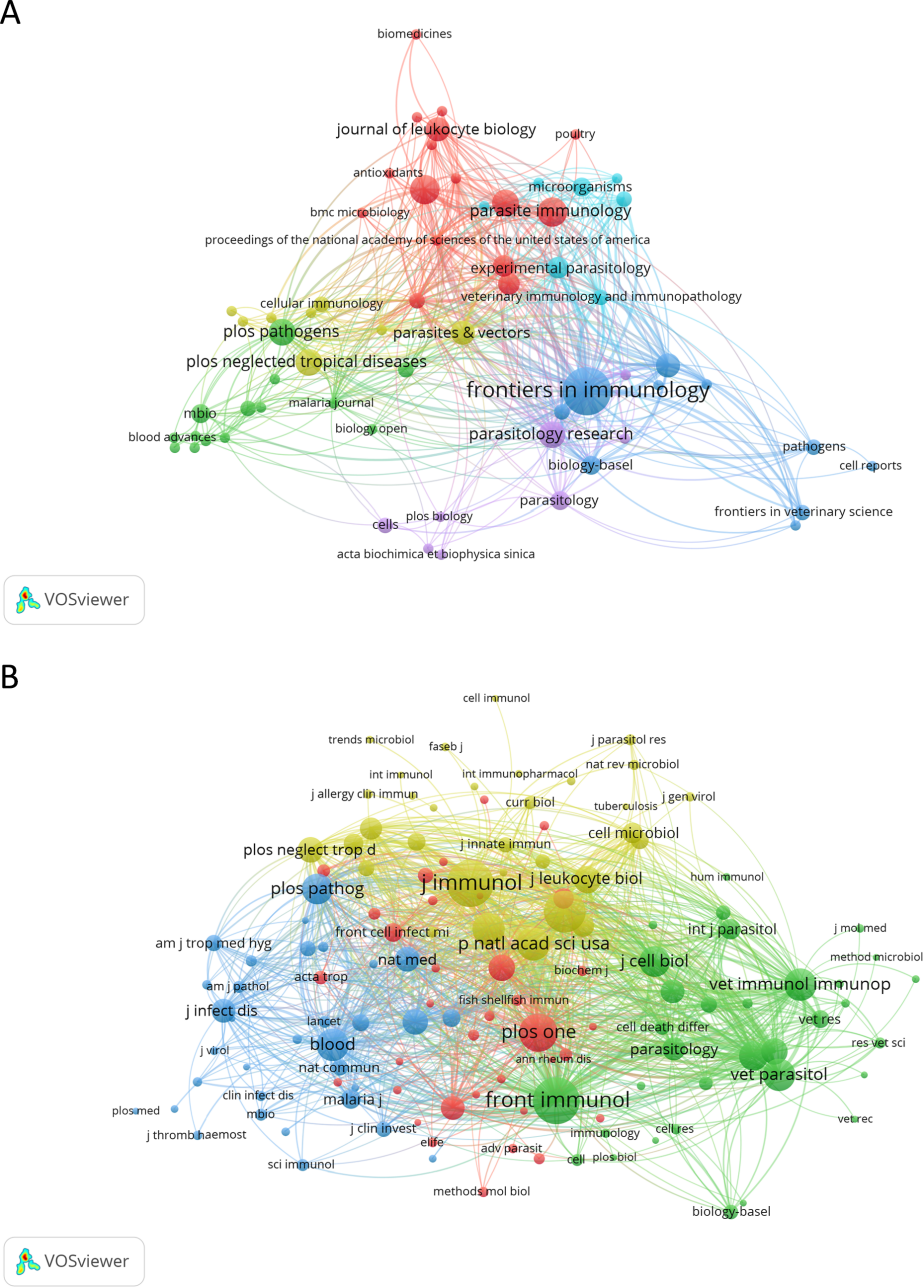
Network map of journals and co-cited journals in the field of NETs and parasites. Node colors represent journals within the same cluster, while node size corresponds to the total number of documents published **(A)** or the total number of co-citations **(B)** for each journal. The analysis in **(B)** includes journals with at least ten citations.

The 159 papers analyzed were published across 69 different journals. Leading the list in terms of the number of documents is *Frontiers in Immunology*, with 19 published papers (11.95%). *Scientific Reports* and *Parasite Immunology* follow, each with 7 papers (4.40%). The most cited journal was *Frontiers in Immunology*, with 477 citations. The *Proceedings of the National Academy of Sciences of the United States of America* ranks second with 438 citations. Notably, this journal has only one published paper in the area, placing it behind 28 other journals that have two or more published papers. *PLoS Pathogens* comes next, with 318 citations. **Figure 5A** and **Supplementary Figure 3** illustrate the journals involved in NETs and parasites research.

The journal impact factor (JIF) reflects the prestige of a journal in a particular field of knowledge. The same is true for the quartiles from the Journal Citation Reports (JCR), which categorize journals according to their ranking within a field (Q1 represents the top 25%, Q2 the next 25%, Q3 the next 25%, and Q4 the bottom 25% of journals in a given research field). Therefore, journals with higher IF and higher quartile rankings are seen as more important than those with lower values. Among the top 10 most productive journals in NETs and parasites, seven (70.0%) belong to Q1, with IFs ranging from 2.7 to 5.7. Two (20.0%) are included in Q2, with IFs of 1.8 and 3.6, and one (10.0%) if from Q3, with an IF of 1.4. *Frontiers in Immunology* (IF = 5.7), *PLoS Pathogens* (IF = 5.5), and *Frontiers in Cellular and Infection Microbiology* (IF = 4.6) stand out as the most influential journals in the field. Among the top 10 journals with the highest number of citations, six (60%) belong to Q1, with IFs varying from 2.7 to 9.4, and four (40%) are included in Q2, with IFs ranging from 1.4 to 3.6. The most prestigious journals with the highest citations in this research area are the *Proceedings of the National Academy of Sciences of the United States of America* (IF = 9.4), *Frontiers in Immunology* (IF = 5.7), and *PLoS Pathogens* (IF = 5.5). **Table 3** and **Supplementary Table 3** present the top 10 journals ranked by the number of articles and the number of citations, respectively.

**Table 3:** Top 10 journals and co-cited journals ranked by the number of articles and the number of co-citations, respectively.

Rank	Journal	Articles	JIF (2024)	JCR	Co-cited journal	Co-citations	JIF (2024)	JCR
1	*Frontiers in Immunology*	19	5.7	Q1	*Journal of Immunology*	393	3.6	Q2
2	*Scientific Reports*	7	3.8	Q1	*Frontiers in Immunology*	391	5.7	Q1
3	*Parasite Immunology*	7	1.4	Q3	*Infection and Immunity*	307	2.9	Q2
4	*PLoS Pathogens*	6	5.5	Q1	*PLoS One*	260	2.9	Q1
5	*Frontiers in Cellular and Infection Microbiology*	6	4.6	Q1	*Proceedings of the National Academy of Sciences of the United States of America*	197	9.4	Q1
6	*PLoS Neglected Tropical Diseases*	6	3.4	Q1	*Veterinary Immunology and Immunopathology*	192	1.4	Q2
7	*Parasitology Research*	6	1.8	Q2	*Science*	191	44.7	Q1
8	*Journal of Leukocyte Biology*	5	3.6	Q2	*Veterinary Parasitology*	189	2.0	Q2
9	*Parasites & Vectors*	5	3.0	Q1	*Blood*	188	21.0	Q1
10	*Developmental and Comparative Immunology*	5	2.7	Q1	*Journal of Cell Biology*	171	7.4	Q1

We also performed a co-citation journal analysis (**Figure 5B**). Co-citation refers to the frequency with which two or more items (e.g., journals, authors, or articles) are simultaneously cited by others. The higher the frequency of co-citation, the stronger their association and the more likely they are to be related within a given field of knowledge. Among the top 10 co-cited journals, four received more than 200 citations, with the *Journal of Immunology* being the most cited (n = 393 co-citations), followed by *Frontiers in Immunology* (n = 391), *Infection and Immunity* (n = 307), and *PLoS One* (n = 260) (**Table 3**). Notably, the most impactful journals on the list of co-cited journals include *Science* (n = 191; IF = 44.7), *Blood* (n = 188; IF = 21.0), and *Proceedings of the National Academy of Sciences of the United States of America* (n = 197; IF = 9.4).

### 3.3 Authors and co-cited authors analysis

**Figure 6:**
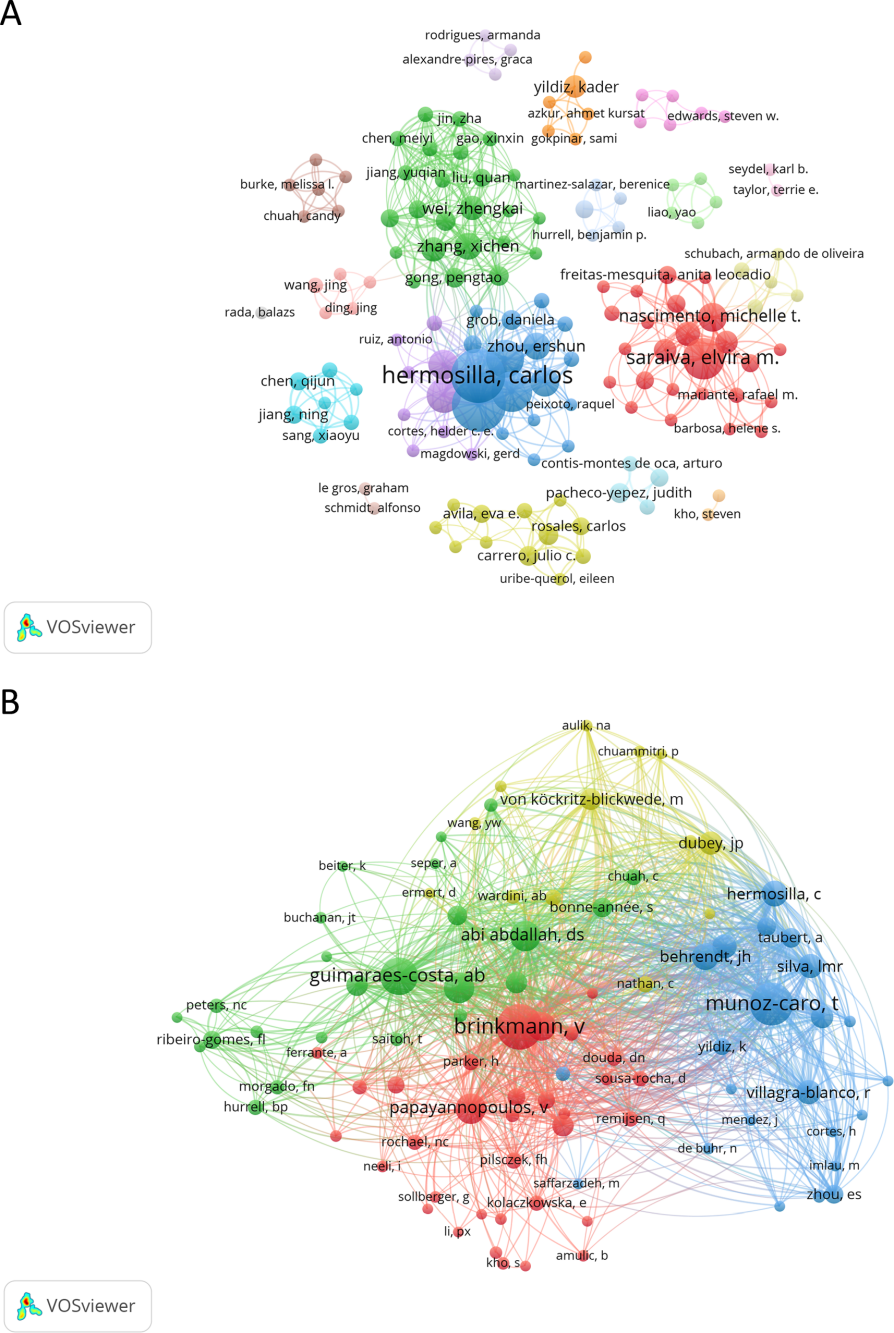
Network map of authors and co-cited authors in the field of NETs and parasites. Node colors represent authors within the same cluster, while node size corresponds to the total number of documents published **(A)** or the total number of co-citations **(B)** for each author. The analysis in **(A)** includes authors with at least two published documents, while in **(B)** it includes authors with ten or more co-citations.

A total of 909 authors contributed to the 159 papers analyzed in this study. The most productive authors are Hermosilla CR (*h*-index of 19) and Taubert A (*h*-index of 19), each with 35 documents. Together, their work accounts for 44.0% of the total production. Following them is Saraiva EM (*h*-index of 12), with 21 papers (13.2%). The list of authors with 10 or more papers also includes Conejeros I (n = 20, 12.6%, *h*-index of 10), Gaertner U (n = 17, 10.7%, *h*-index of 11), Munoz-Caro T (n = 14, 8.8%, *h*-index of 12), and Silva LMR (n = 11, 6.9%, *h*-index of 9). The most frequently cited author is Saraiva EM, with 1,070 citations, followed by Hermosilla CR and Taubert A, each with 995 citations. Guimaraes-Costa AB (*h*-index of 6) and Nascimento MTC (*h*-index of 8) are also highly cited, with 898 and 850 citations, respectively. **Figure 6A** and **Supplementary Figure 4** show the authors involved in research on NETs and protozoan or helminths parasites and their collaborative network.

Our co-citation author analysis revealed that out of a total of 4,415 co-cited authors, 38 have been cited at least 20 times. Brinkmann V (n = 180) was the most frequently co-cited author, followed by Munoz-Caro T (n = 159) and Guimaraes-Costa AB (n = 121). Among the co-cited authors with 60 or more citations are Abi Abdallah DS (n = 82), Papayannopoulos V (n = 82), Urban CF (n = 80), Fuchs TA (n = 71) and Behrendt JH (n = 64), (**Figure 6B**). **Table 4** presents the top 10 authors and co-cited authors ranked by the number of original articles and citations, respectively. **Supplementary Table 4** presents the top 10 most prolific authors in the area ranked by the number of citations.

**Table 4:** Top 10 authors and co-cited authors ranked by the number of original articles and co-citations, respectively.

Rank	Author	Articles	Citations	*h*-Index^*^	Co-cited author	Co-citations
1	Hermosilla CR	35	995	19	Brinkmann V	180
2	Taubert A	35	995	19	Muñoz-Caro T	159
3	Saraiva EM	21	1,070	12	Guimarães-Costa AB	121
4	Conejeros I	20	324	10	Abi Abdallah DS	82
5	Gaertner U	17	387	11	Papayannopoulos V	82
6	Muñoz-Caro T	14	609	12	Urban CF	80
7	Silva LMR	11	340	9	Fuchs TA	71
8	Nascimento MTC	10	850	8	Behrendt JH	64
9	Velasquez ZD	10	124	7	Hermosilla CR	59
10	Zhang X	9	187	6	Silva LMR	52

*The *h*-index reported here is calculated based solely on the papers included in this analysis, reflecting the authors’ impact within the scope of the study.

### 3.4 References and co-cited references analysis

**Figure 7:**
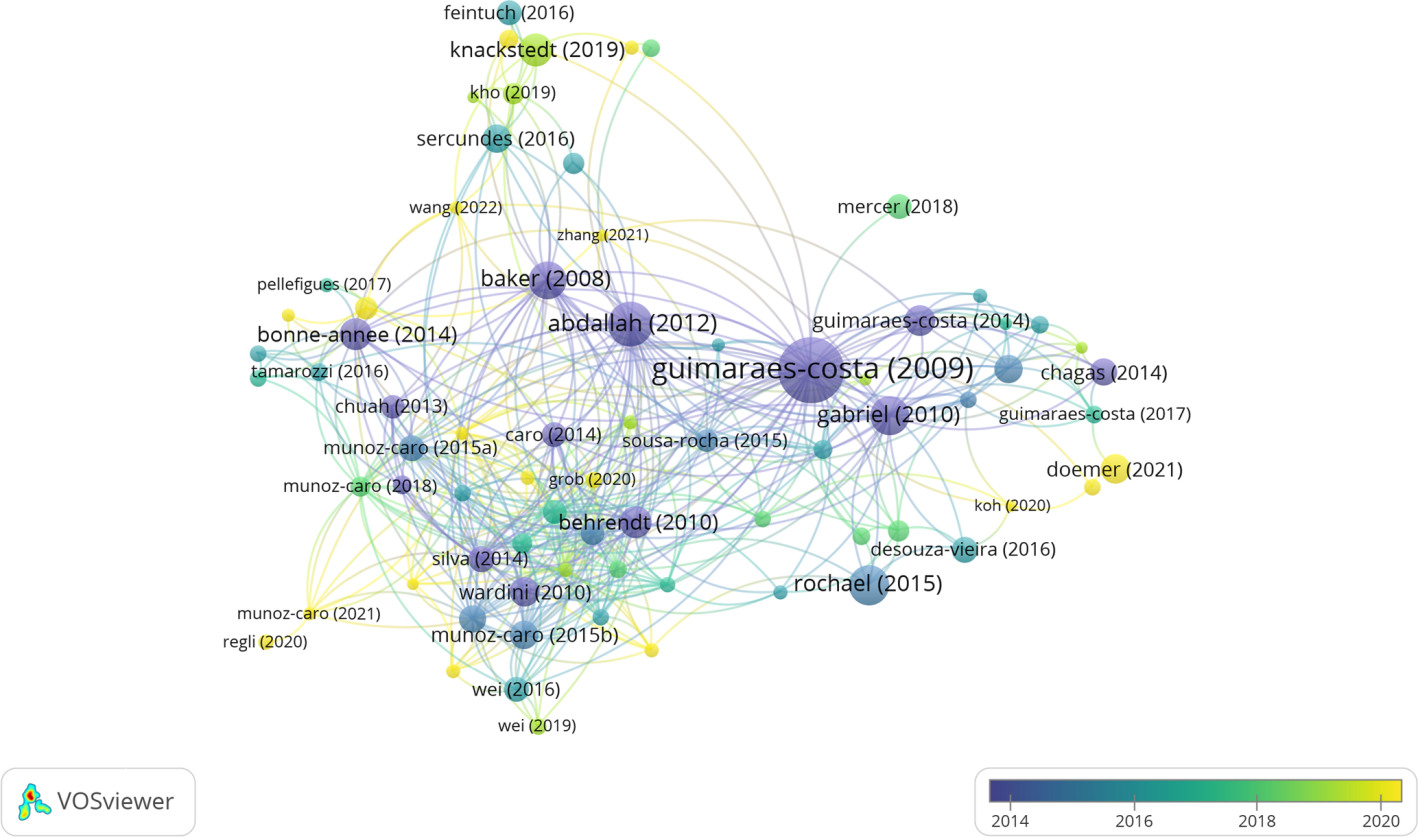
Network map analysis of the most frequently cited articles. This analysis includes articles with at least fifteen citations. Node colors represent the average publication year of the papers, while node sizes indicate the number of citations for the corresponding documents.

The most frequently cited article in the research area is “*Leishmania amazonensis* promastigotes induce and are killed by neutrophil extracellular traps” (16) with 438 citations. Notably, this paper was the first to show direct evidence of NETs triggered by a protozoan parasite, *Leishmania*. The second most highly cited paper is “*Toxoplasma gondii* Triggers Release of Human and Mouse Neutrophil Extracellular Traps” (17) with 201 citations, which describes the release of NETs induced by *Toxoplasma*, another protozoan. In the list of papers with 100 or more citations, there are also two more papers on *Leishmania*, depicting the mechanisms and molecules involved in NETs production (18; 165 citations; 19; 156 citations); two papers on *Plasmodium* (20; 145 citations; 21; 113 citations), the former being the first-ever published paper on NETs and parasites, in which the authors identified the presence of NETs in the blood of children with malaria; one paper on *Eimeria*, a close relative of *Toxoplasma* (22; 106 citations); and one paper on the nematode *Strongyloides* (23; 105 citations), the most cited paper in the field of NETs and helminth parasites. **Figure 7** shows the network of the most frequently cited articles, and **Table 5** shows the top 10 highly cited articles in the research area.

**Table 5:** Top 10 highly cited original articles in the research field ranked by the number of citations.

Rank	Title	Author/Year	Journal	Citations
1	*Leishmania amazonensis* promastigotes induce and are killed by neutrophil extracellular traps	Guimarães-Costa et al., 2009 (16)	*Proc Natl Acad Sci USA*	438
2	*Toxoplasma gondii* Triggers Release of Human and Mouse Neutrophil Extracellular Traps	Abi Abdallah et al., 2012 (17)	*Infect Immun*	201
3	Classical ROS-dependent and early/rapid ROS-independent release of Neutrophil Extracellular Traps triggered by *Leishmania* parasites	Rochael et al., 2015 (18)	*Sci Rep*	165
4	*Leishmania donovani* promastigotes evade the antimicrobial activity of neutrophil extracellular traps	Gabriel et al., 2010 (19)	*J Immunol*	156
5	Cytokine-associated neutrophil extracellular traps and antinuclear antibodies in *Plasmodium falciparum* infected children under six years of age	Baker et al., 2008 (20)	*Malar J*	145
6	Neutrophil extracellular traps drive inflammatory pathogenesis in malaria	Knackstedt et al., 2019 (21)	*Sci Immunol*	113
7	Neutrophil extracellular trap formation as innate immune reactions against the apicomplexan parasite *Eimeria bovis*	Behrendt et al., 2010 (22)	*Vet Immunol Immunopathol*	106
8	Extracellular traps are associated with human and mouse neutrophil and macrophage mediated killing of larval *Strongyloides stercoralis*	Bonne-Année et al., 2014 (23)	*Microbes Infect*	105
9	3′-nucleotidase/nuclease activity allows *Leishmania* parasites to escape killing by neutrophil extracellular traps	Guimarães-Costa et al., 2014 (24)	*Infect Immun*	99
10	Neutrophil Extracellular Traps Activate Proinflammatory Functions of Human Neutrophils	Dömer et al., 2021 (25)	*Front Immunol*	93

The analysis of co-cited references revealed the original description of NETs by Brinkmann et al. (1) as the most co-cited article (n = 116), followed by the paper from Guimaraes-Costa et al. (16) describing NETs triggered by *L. amazonensis* (n = 75). Additionally, the top 10 list of co-cited references with more than 50 citations includes the paper by Fuchs et al. (26), which details the sequence of events that culminate in NETs release and the involvement of ROS generated by NADPH oxidase (n = 67), and the paper by Abi Abdallah et al. (17), which depicts NETs induced by *T. gondii* (n = 59). **Supplementary Table 5** lists the top 10 co-cited references in NETs and parasites.

We also investigated the top 25 references with the strongest citation bursts, a tool from CiteSpace used to reveal the hot co-cited references with an abrupt increase in citations over time. Many papers published in NETs and parasites have been frequently cited in recent years, indicating that research in this field is likely to continue growing in the future. Published in the *Journal of Immunology*, the article “*Leishmania donovani* promastigotes evade the antimicrobial activity of neutrophil extracellular traps” by Gabriel et al. (19) had the strongest citation burst (strength = 6.74), which occurred between 2012 and 2015. The paper from Abi Abdallah et al. (17) published in *Infection and Immunity*, entitled “*Toxoplasma gondii* triggers release of human and mouse neutrophil extracellular traps”, also had a strong burst (strength = 6.55) from 2014 to 2017 (**Supplementary Figure 5**). Notably, several publications are currently within the citation burst phase, indicating that research on the subject is likely to continue growing in the coming years.

### 3.5 Analysis of keywords, trend topics and thematic map

#### 3.5.1 Keywords

**Figure 8:**
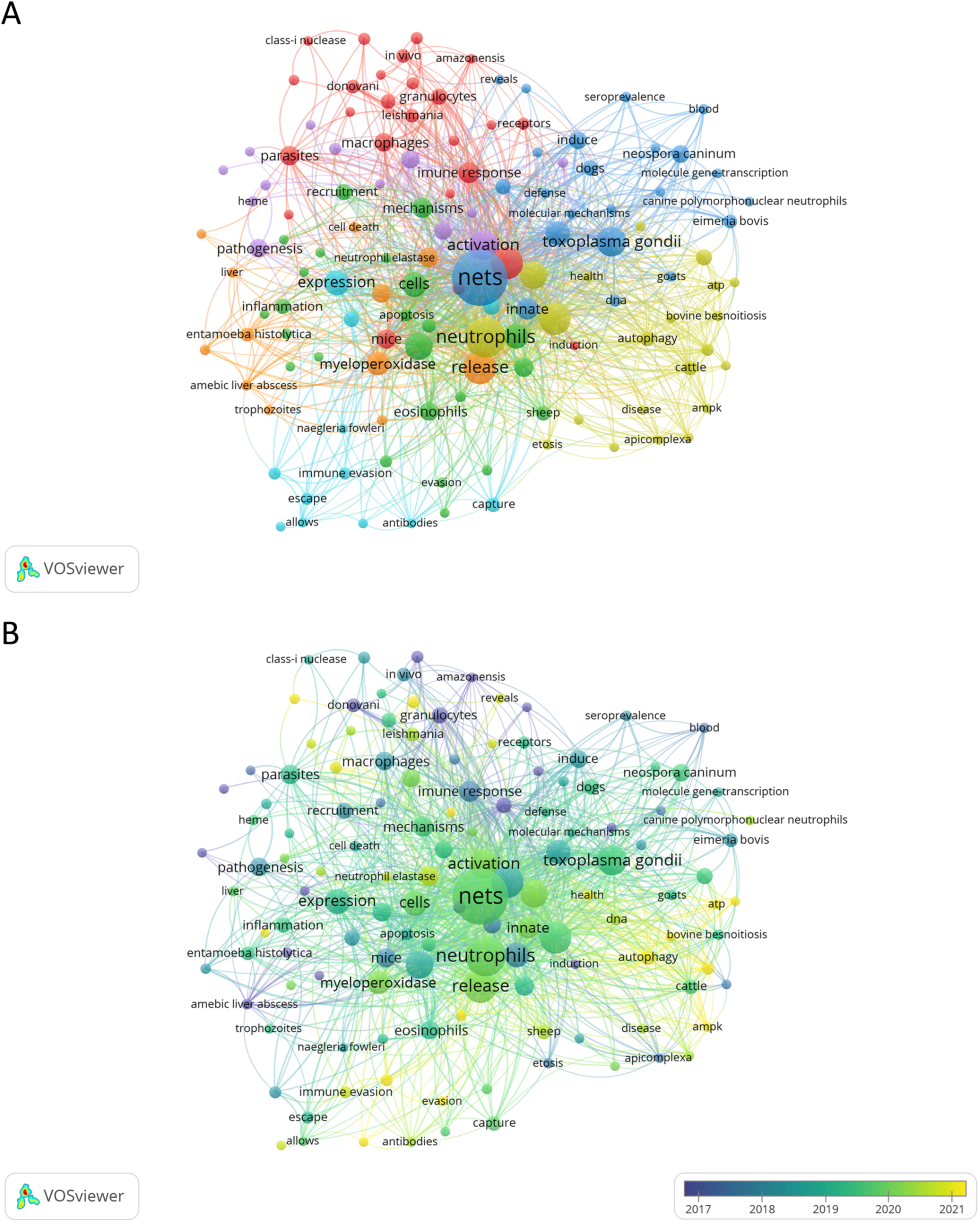
Network map analysis of the most frequent keywords related to NETs and parasites. Node colors represent keywords within the same cluster **(A)** or the average publication year of the keywords **(B)**. Node sizes indicate the total occurrences of each keyword in both analyses **(A, B)**. The analyses include keywords with three or more occurrences.

The analysis of keywords allows for the summarization of study topics in a specific research area, enabling the exploration of hotspots and directions within that area. For this analysis, we first manually combined keywords that were synonyms or that appeared in singular or plural forms. For example, “*neutrophil extracellular traps*”, “*neutrophils extracellular traps*”, “*neutrophil extracellular trap*”, “*neutrophil extracellular traps (net)*”, “*neutrophil extracellular traps (nets)*”, “*nets (neutrophil extracellular traps)*”, “*extracellular trap*”, “*extracellular traps*”, “*extracellular dna traps*”, and “*net*” were combined into the keyword “*NETs*.” This resulted in a total of 776 keywords, which were then analyzed for their frequency of appearance. VOSviewer was utilized to compute keyword occurrences (incorporating both Author Keywords and Keywords Plus) and Biblioshiny to generate a word cloud based on the Keywords Plus list. The most frequently cited keyword was *NETs*, with 98 occurrences, followed by *neutrophils* (n = 52) and *release* (n = 35). Other keywords with 25 or more occurrences included *infection* (n = 33), *NETosis* (n = 32), *Toxoplasma gondii* (n = 29), *activation* (n = 27), *innate immunity* (n = 25), and *immunity* (n = 25). **Supplementary Table 6** lists the top 20 most frequently cited keywords. The co-occurrence network of keywords was divided into various clusters identified by different colors, reflecting either the basic knowledge structure of related research fields (**Figure 8A**) or the average publication year of a given keyword (**Figure 8B**). Using the WordCloud tool from Biblioshiny, we generated a word cloud of the 50 most frequently used research keywords in the study of NETs and parasites (**Supplementary Figure 6**).

#### 3.5.2 Trend topics

**Figure 9:**
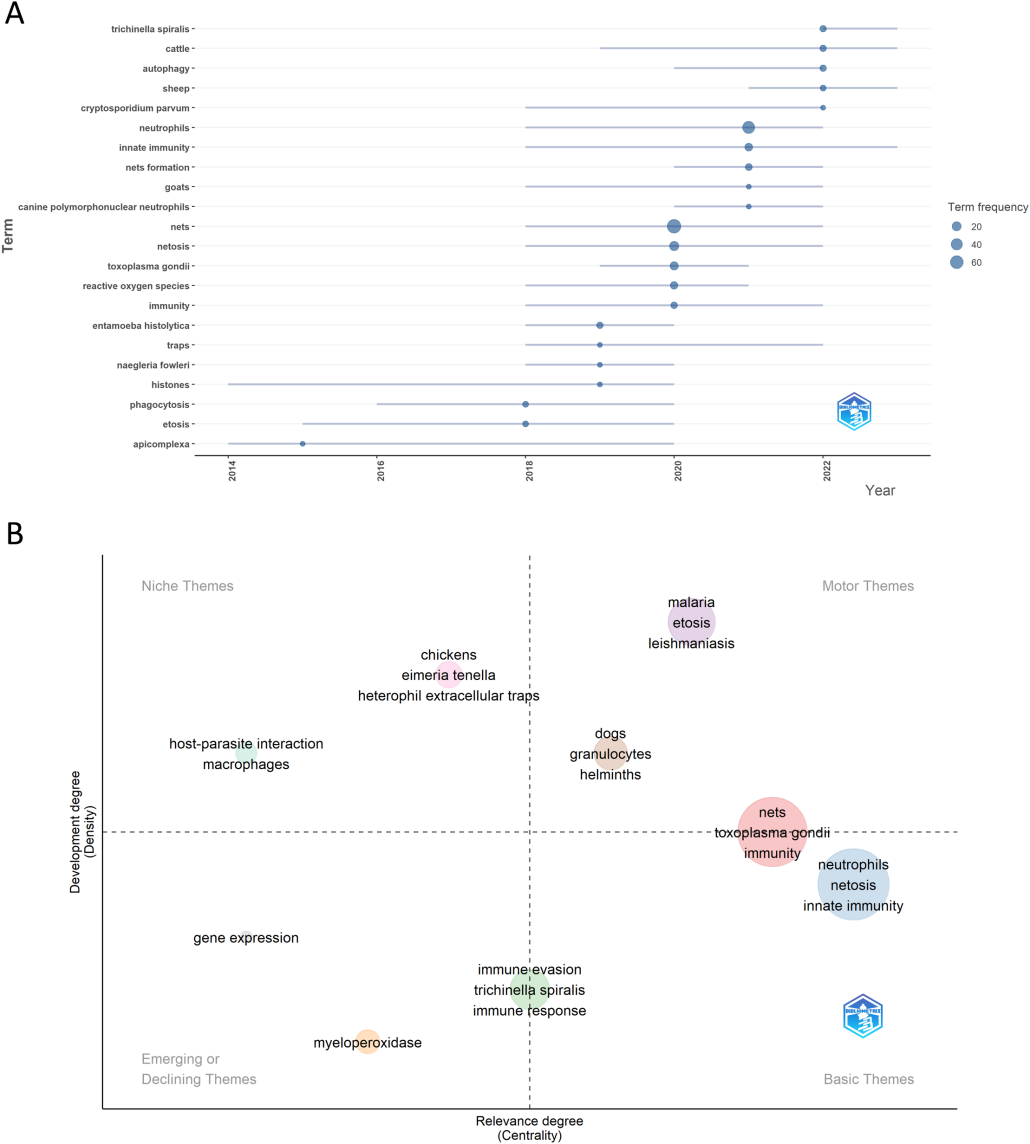
Trend topics and thematic map in the field of NETs and parasites. **(A)** highlights the trend topics derived from the authors’ keywords for the analyzed period, while **(B)** displays the key motor, basic, emerging/declining, and niche themes also identified from the authors’ keywords.

To understand past and emerging research directions, we analyzed current research hotspots and future trends (**Figure 9A**). In general, we identified three distinct periods of development: The first period (2014-2020) included keywords such as *histones*, *phagocytosis*, *ETosis*, and *Apicomplexa*, reflecting a focus on neutrophils in immune responses, particularly in Apicomplexa parasites. Research explored neutrophil-mediated mechanisms and the role of histones, highlighting an interest in neutrophils’ contribution to defense against *Plasmodium*, *Eimeria*, *Toxoplasma*, and *Besnoitia* infections. The second period (2018-2022) featured high-frequency keywords such as *Cryptosporidium*, *Toxoplasma*, *Entamoeba*, *Naegleria*, *ROS*, *NETs*, and host-specific immune responses (e.g., *canine neutrophils*, *goats*, and *innate immunity*). This period shows an expanding focus on neutrophils’ role in immune defense through NET formation against a range of protozoan pathogens, along with variations in immune responses across host species. Finally, the period from 2020 to the present includes keywords like *Trichinella*, *cattle*, *autophagy*, and *sheep*. This suggests a shift toward studying helminth infections and understanding how parasites modulate immune responses in livestock. Additionally, the focus on livestock points to a practical approach aimed at understanding and mitigating the effects of parasitic infections on livestock health. Keywords like *autophagy* suggest that the immune response to the parasite may involve processes beyond NET release, with different mechanisms potentially occurring simultaneously.

#### 3.5.3 Thematic map

A thematic map provides a visual representation of the progression and importance of research themes by analyzing two key metrics: density, which reflects the theme’s internal development and coherence, and centrality, which measures its relevance and connectivity to other themes within the broader research field (14). In the thematic map of NETs and parasites, distinct clusters provide insight into the research focus and evolution within the field (**Figure 9B**). Motor themes, which are central, highly developed, include keywords such as *malaria*, *ETosis*, *leishmaniasis*, *dogs*, *granulocytes*, and *helminths*. These indicate established areas of active investigation, with strong relevance to the field. Basic themes, representing foundational concepts, are characterized by keywords like *neutrophils*, *netosis*, and *innate immunity*, emphasizing the core elements underpinning studies of NETs and parasitic interactions. Emerging or disappearing themes include *myeloperoxidase* and *gene expression*, which may reflect novel or declining areas of interest. Niche themes, focused on more specialized topics, encompass *host-parasite interaction*, *macrophages*, *chickens*, *Eimeria tenella*, and *heterophil extracellular traps*. These themes suggest research concentrated on specific host-parasite systems or particular immune mechanisms. Keywords like *NETs*, *Toxoplasma gondii*, and *immunity* occupy a transitional space between motor and basic themes, indicating their dual role as fundamental to the field and experiencing active development. In contrast, terms like *immune evasion*, *Trichinella spiralis*, and *immune response* lie between basic and emerging/declining themes, suggesting they are foundational but may be in flux—either gaining prominence or losing relevance.

## 4 Discussion

Following the first publication on NETs two decades ago by Brinkmann et al. (1), researchers worldwide have concentrated their efforts on unraveling the mechanisms behind extracellular trap production in response to various stimuli. While a few recent studies have presented bibliometric data on NETs in general (27, 28) or in the context of autoimmune conditions (29, 30) and tumors (31), bibliometric reviews focused on NETs and infectious agents are lacking. In this study, we employed various scientific tools to analyze the data and create comprehensive visual maps of 159 original articles on NETs triggered by parasites, published between 2008 and 2024, identifying potential trends and hotspots in the field.

### 4.1 General information

Our analysis reveals a rapid increase in the number of original articles published on this subject. Between 2008 and 2013, the output was about one paper annually, rising to more than nine per year from 2014 to 2018 and exceeding 17 annually from 2019 to 2024. The number of publications peaked in 2021 and 2024, with 20 papers each. This consistent growth in research output, coupled with recent citation bursts, indicates that NETs and parasites are likely to remain a significant focus of research in the near future. An analysis of country contributions shows Germany, Brazil, and China as the leading nations in this research area. Interestingly, these findings contrast with broader bibliometric studies on NETs, such as those not focused on specific topics (27, 28), or NETs related to autoimmune diseases (29, 30) and tumors (31), where the USA, China, and Germany lead. Nevertheless, the emergence of Brazil as a leader in NETs and parasites is not surprising, given the longstanding contributions of the Brazilian research community to parasitology. For over 100 years, Brazilian scientists have studied parasites and their vectors, including protozoans and helminths, many of which are endemic to the country, such as those causing Chagas disease, malaria, leishmaniasis, and schistosomiasis, though most remain neglected (32–34). Significant milestones in protozoan research were achieved in Brazil in the early 20th century, including Carlos Chagas’s ‘triple discovery’ (pathogen, vector, and disease) of *T. cruzi* (35), the first visualization and description of *T. gondii* by the Italian researcher Alfonse Splendore, who was based in Brazil (36), and Gaspar de Oliveira Vianna’s description and naming of *Leishmania braziliensis* (37). Furthermore, a recent bibliometric analysis covering a 30-year period (1989–2019) highlighted Brazil’s substantial contributions to parasitology, encompassing studies on a wide range of helminth and protozoan infections, parasites of veterinary importance, and the treatment of parasitic diseases (38).

Among the top 10 most productive institutions, Justus Liebig University Giessen in Germany, led by Carlos Hermosilla and Anja Taubert, and the Federal University of Rio de Janeiro in Brazil, led by Elvira Saraiva, are primarily responsible for the prominent positions these countries have achieved. Notably, Brazil also has two other institutions in the top 10 most productive list—the Oswaldo Cruz Foundation (Fiocruz) and the University of São Paulo—further highlighting the country’s strong interest and leadership in this growing research area. Most publications on this topic appear in *Frontiers in Immunology*, which also holds the highest number of total citations. Among the journals with the most articles, the majority are rated JRC Qualis Q1, underscoring the recognized importance of the field. Interestingly, the journal with the highest number of citations per article, *Proceedings of the National Academy of Sciences of the United States of America*, ranks only 29th among the most productive journals, having published just one paper on this subject. Remarkably, the paper by Anderson Guimarães-Costa et al. (16), published in this journal, which describes the release of NETs in response to *Leishmania amazonensis*, has received nearly as many citations as all 17 papers published in *Frontiers in Immunology* combined, underscoring its significant impact. While the malaria paper by Virginia Baker et al. (20) was the first to demonstrate the presence of NETs in the context of a parasitic infection, it relied on indirect evidence and did not conclusively show that parasites alone could induce NETs release. Guimaraes-Costa’s paper was the first to demonstrate that neutrophils directly trigger NETs formation in response to parasites and that NETs can kill these microorganisms.

Carlos Hermosilla and Anja Taubert are the most productive authors, ranking second in citation frequency. Elvira Saraiva is the most cited author, ranking behind Hermosilla and Taubert in the number of original publications. The German group’s most-cited article, “Neutrophil extracellular trap formation as innate immune reactions against the apicomplexan parasite *Eimeria bovis*” (2010), ranks seventh on the top 10 list. The Brazilian team’s work has the greatest impact in the field, with “*Leishmania amazonensis* promastigotes induce and are killed by neutrophil extracellular traps” (2009) and “Classical ROS-dependent and early/rapid ROS-independent release of Neutrophil Extracellular Traps triggered by *Leishmania* parasites” (2015) ranking first and third, respectively, and “3′-nucleotidase/nuclease activity allows *Leishmania* parasites to escape killing by neutrophil extracellular traps” (2014) ranking nineth. Remarkably, the paper “*Toxoplasma gondii* triggers release of human and mouse neutrophil extracellular traps” (2012) by Eric Denkers’ team in the USA ranks second on the top 10 list and also has a significant influence in the field of NETs and parasites. The most co-cited author is Volker Brinkmann, with his seminal work “Neutrophil extracellular traps kill bacteria” (2004) topping the list of highest co-cited references. Notably, Brinkmann has also contributed to the field of NETs and parasites with the publication “Neutrophil extracellular traps drive inflammatory pathogenesis in malaria” (2019), which ranks sixth on the top 10 list of most cited papers. This study was published in *Science Immunology*, the journal with the highest impact factor (IF = 17.6) among those listed.

### 4.2 Hotspots and frontiers

The analysis of keywords, trend topics, thematic map. and papers with the strongest citation bursts highlights current trends and hotspots in the study of NETs triggered by parasites. As shown by the trend topic and thematic analyses, over the years, an increasing number of papers have focused on NETs triggered by a wide diversity of protozoa and helminths, as well as on the mechanisms involved in NETs release and the characterization of NETs produced by different host species. Studies on the protozoa *Plasmodium*, *Leishmania*, *Toxoplasma*, and the nematode *Strongyloides*, published between 2008 and 2015, paved the way for the consolidation of this field of research. Publications on specific host-parasite systems, such as those involving various species of *Eimeria* and their hosts, offer valuable insights into the evolution of the innate immune response within these systems. For example, viable *E. bovis* sporozoites and *E. arloingi* sporozoites and oocysts induced the release of NETs in bovine and caprine neutrophils, respectively (22, 39). These NETs trap and immobilize infective parasitic stages, reducing their ability to infect host cells. The severe pathogen *E. ninakohlyakimovae*, which causes hemorrhagic typhlocolitis in goats, also induced NET formation, which in turn reduced epithelial invasion by sporozoites, lowered macromeront formation, and subsequently decreased merozoite production (40). Additionally, NET components have been detected near various *Eimeria* stages and within the gut lumen of coccidia-infected caprines and bovines, where they interacted with released oocysts (41). This suggests a stage-independent mechanism of NET extrusion *in vivo*. Studies in chickens have shown that *E. tenella* sporozoites induce the formation of heterophil extracellular traps (HETs) (42), further emphasizing the parasite stage-independent heterophil responses to this pathogenic coccidian (43).

Recently, significant research focused on NETs induced by the apicomplexan *Plasmodium*, *Toxoplasma gondii* and *Besnoitia besnoiti*, as well as the mechanisms of their formation, release, and impact on parasites and host cells. NETs triggered by nematodes and their immune evasion mechanisms are also key topics. Below, we outline major trends and future research directions based on recent studies with notable citation bursts.

#### 4.2.1 Protozoa

In the study “Neutrophil extracellular traps drive inflammatory pathogenesis in malaria,” Knackstedt et al. (21) explored the role of NETs in malaria immunopathology using human samples and murine models. The study identifies heme, a byproduct of malaria-induced hemolysis, as a key trigger for NET release. NETs promote inflammation and vascular pathology, upregulating endothelial cytoadherence receptors that facilitate parasite sequestration in vital organs like the liver and lungs. Disrupting NET formation significantly reduced organ damage in infected mice, highlighting NETs as therapeutic targets, though they did not harm parasites in the *P. chabaudi* mouse model. Rodrigues et al. (44) also examined NET release during *Plasmodium* infection, showing that infected red blood cells release macrophage migration inhibitory factor (MIF), which induces NET formation via CXCR4 interaction. NETs reduced infected erythrocytes and restrained parasite spread, as evidenced by increased parasitemia and mortality after NET disruption in a *P. berghei ANKA* cerebral malaria model. In human malaria caused by *P. falciparum, P. vivax*, and *P. malariae*, an increase in circulating NETs was observed, with the highest levels found in patients with severe disease (45). These findings establish NETs not only as biomarkers of disease severity but also as active drivers of the pathological sequelae in malaria, suggesting that adjunctive therapies targeting NETs could mitigate severe malaria complications.

In the paper “Simultaneous and positively correlated NET formation and autophagy in *Besnoitia besnoiti* tachyzoite-exposed bovine polymorphonuclear neutrophils,” researchers investigated the interaction between bovine neutrophils and the parasite *B. besnoiti*, responsible for bovine besnoitiosis (46). The study highlights how *B. besnoiti* tachyzoites trigger both NET formation and autophagy in neutrophils, confirming that these phenomena occur simultaneously in bovine neutrophils, with LC3B-related autophagosome formation and AMP-activated protein kinase α (AMPKα) phosphorylation, suggesting a connection between the two mechanisms. While the paper establishes the coexistence of autophagy and NETs formation in response to the parasite, future studies should aim to unravel the precise molecular mechanisms that independently regulate these pathways. This could pave the way for novel therapeutic approaches to control bovine besnoitiosis. Since autophagy and NETs formation are not unique to bovines (47–49), exploring whether similar mechanisms are present in other host species infected by related parasites could extend the relevance of these findings to both human and veterinary medicine.

Another study that explored *B. besnoiti*’s ability to induce NETs release from bovine neutrophils is “Histone H2A and bovine neutrophil extracellular traps induce damage to *Besnoitia besnoiti*-infected host endothelial cells but fail to affect total parasite proliferation” (50). The study found that while NETs cause damage to infected host endothelial cells *in vitro*, they do not significantly impact the parasite’s overall proliferation. This suggests that the parasite has developed mechanisms to evade or resist the antimicrobial effects of NETs, allowing it to persist in the host despite a damaging immune response. Given the dual role of NETs in inflicting damage on host cells while failing to reduce total parasite proliferation, future research could explore therapeutic strategies to modulate NETs formation, minimizing host damage while enhancing antiparasitic efficacy. Such advances could lead to improved control strategies for bovine besnoitiosis.

The article “Dolphin-derived NETosis results in rapid *Toxoplasma gondii* tachyzoite ensnarement and different phenotypes of NETs” demonstrated how dolphin neutrophils form NETs with distinct phenotypes that rapidly ensnare *T. gondii* tachyzoites in a ROS-dependent manner (51). The findings revealed three NET phenotypes—spread, diffuse, and aggregated—with spread and aggregated NETs being more frequently induced by tachyzoites. Additionally, the study analyzed ‘cell-free’ and ‘anchored’ dolphin NETs, showing that tachyzoite exposure significantly increased the formation of ‘anchored’ NETs, while ‘cell-free’ NET formation remained largely unaffected. These findings suggest a species-specific immune defense mechanism in marine mammals. Future research should investigate how NETosis varies across marine and terrestrial mammals, particularly in response to protozoan pathogens. Understanding whether these differences stem from ecological factors, pathogen exposure, or evolutionary pressures could provide insights into the immune mechanisms of marine mammals. Given the substantial significance of *T. gondii* as a parasite in marine animals (52–54), further studies should examine its impact on dolphin populations and other marine species, as well as how these animals have evolved to combat tissue-cyst-forming parasites. Exploring the molecular pathways driving different NET phenotypes could offer new insights into immune responses and lead to novel therapeutic approaches to enhance NET function in both human and animal health.

The study “*Trypanosoma brucei brucei* induces polymorphonuclear neutrophil activation and neutrophil extracellular traps release” demonstrates that exposure to *T. b. brucei* trypomastigotes activates neutrophils, leading to enhanced oxygen consumption, ROS production, and the formation of various NET phenotypes, which are dependent on purinergic signaling (55). These NETs ensnare the parasites but do not significantly impact their viability, although they do reduce parasite motility. The role of NETs in exacerbating intravascular coagulation and vascular permeability during trypanosomiasis warrants further investigation. Understanding these mechanisms could provide insights into mitigating disease severity, particularly in livestock. Moreover, targeting purinergic signaling might offer new therapeutic strategies by modulating NET formation and reducing the pathological consequences of infection. Expanding this research to other trypanosomes, or comparing NET responses in human African trypanosomiasis, could enhance our understanding of species-specific immune responses and provide deeper insights into host-parasite interactions.

#### 4.2.2 Helminths

The study “*Dirofilaria immitis* microfilariae and third-stage larvae induce canine NETosis resulting in different types of neutrophil extracellular traps” (2018) demonstrates how the parasitic nematode *Dirofilaria immitis*, which causes heartworm disease in dogs and pulmonary or cutaneous infections in humans, triggers different types of NETs (56). Specifically, microfilariae stages induce spread and diffuse NETs, while the larger third-stage larvae (L3) additionally trigger aggregated NETs formation. These stages promote significant NETs entrapment without killing the larvae, and treatment with the NADPH oxidase inhibitor diphenyleneiodonium did not significantly alter these reactions. This suggests a complex interaction between the parasite and the host’s immune system concerning NETs formation. The research highlights the distinct NETs types acting against various stages of *D. immitis*, but leaves open the question of the specific signaling pathways involved in this process. Future studies could uncover the molecular signals that trigger specific NETs types in response to this parasite. Unlike most parasitic nematodes, *D. immitis* stages inhabit the right heart and blood vessels, exposing them to a challenging environment composed of innate and adaptive immune cells (e.g., neutrophils, monocytes, T cells, and NK cells), complement factors, antibodies, and cytokines/chemokines. The release of NETs in these environments may contribute to intravascular damage, affecting disease pathogenesis and outcomes. Given that excessive or dysregulated NETosis has been linked to autoimmune diseases and tissue damage (57), these findings suggest a potential therapeutic avenue in modulating NETs formation for heartworm disease. While this study focuses on canine models, similar research in other species affected by the parasite, such as humans and wildlife, may reveal host-specific immune responses to *D. immitis* and other filarial parasites, offering new insights into host-pathogen interactions in dirofilariasis.

In the paper “Hookworms evade host immunity by secreting a deoxyribonuclease to degrade neutrophil extracellular traps,” the investigators demonstrate how *Nippostrongylus brasiliensis* and *Necator americanus*, which are responsible for murine and human hookworm diseases, respectively, induce NETs formation that contributes to the killing of hookworm larvae *in vitro* and limits parasite viability *in vivo* (58). However, the study found that hookworms secrete a deoxyribonuclease enzyme (Nb-DNase II) that degrades the DNA backbone of NETs, allowing the larvae to evade NET-mediated killing and escape the immune response. Neutralizing Nb-DNase II enhanced the ability of neutrophils to kill larvae *in vitro*, suggesting potential avenues for developing vaccines or immune therapies targeting Nb-DNase II. This could improve immune effectiveness and limit hookworm infections. Additionally, DNase activity has been identified in the excretory and secretory products of other helminths, including *Trichinella spiralis* and *Haemonchus contortus* (59, 60), indicating that this mechanism may be widespread among parasitic species. Future research could explore this mechanism across a broader range of parasites to better understand the prevalence of NETs evasion strategies.

### 4.3 Implications for global health policies and parasite control

Understanding how protozoa and helminths induce NET release, evade NET-mediated defenses, and contribute to chronic infections has profound implications for public health and parasite control strategies. Clarifying the mechanisms behind NET formation could pave the way for targeted interventions, including immunomodulatory therapies and enhanced diagnostics. NETs have been observed during various parasitic infections, such as malaria (20), leishmaniasis (16), and in the liver during infections caused by *Schistosoma japonicum* (61), *Opisthorchis viverrini* (62), or *Fasciola hepatica* (63). These findings suggest a dual role of neutrophils in the progression of protozoan and trematode-borne diseases, where excessive NET production may lead to organ complications, including hepatobiliary abnormalities and conditions like cholangiocarcinoma (62). Such observations emphasize the need to balance the protective and pathological roles of NETs in parasitic infections, opening new research and therapeutic avenues.

Studies on NET release in host defense and disease pathogenesis have already prompted the exploration of NET inhibitors in conditions like autoimmune diseases, cancer, and sepsis (64). Translating these approaches to parasitic diseases could mitigate NET-induced inflammation, as seen in cerebral malaria. In leishmaniasis, NET modulation may enhance treatment outcomes by reducing tissue damage while preserving parasite clearance. Combining antiparasitic drugs with NET inhibitors in parasite control programs could refine strategies such as vaccination and treatments, addressing NET-mediated complications and improving overall treatment efficacy. Understanding the role of NETs in parasitic diseases is crucial for enhancing health strategies and global parasite control. However, further research is needed to clarify their roles in diverse parasitic conditions.

## 5 Limitations

Using visual tools and bibliometric methods, we have, for the first time, comprehensively analyzed the research trends and developments of NETs triggered by parasites since 2008, when the first paper on the subject was published. However, our study has some limitations. First, our analysis did not assess the quality of the publications, so we lack an adequate measure of article quality. Consequently, our findings should be interpreted as indicative of research trends and outputs rather than as a critique of the studies’ quality. Second, the search was conducted on December 7, 2024, which may have resulted in an incomplete dataset for that year. Finally, we manually merged synonyms for countries, journals, keywords, authors, institutions, co-cited authors, and co-cited references, which could have introduced some artificial bias.

## 6 Conclusion

In this study, we used bibliometrics to review publications on NETs and parasites, identifying trends and hotspots in the field. Germany and Brazil have emerged as leading powers in this area of research. Carlos Hermosilla and Anja Taubert, from Justus Liebig University Giessen, and Elvira Saraiva, from the Federal University of Rio de Janeiro, are recognized as authoritative figures in the subject. Malaria, *Toxoplasma gondii*, *Besnoitia besnoiti*, and nematodes are prominent topics within the field. Deciphering the mechanisms behind NETs release, understanding the effects of NETs on parasite and host cell viability, therapeutic targeting of NETs, and conducting comparative studies across different host and parasite species could provide valuable directions for future research. We hope this study will assist researchers in gaining a clearer understanding of the current trends in the field.

## Data Availability

The original contributions presented in the study are included in the article/**Supplementary Material**. Further inquiries can be directed to the corresponding author.
