# Supplementary material for: Bibliometric analysis of neutrophil extracellular traps induced by protozoan and helminth parasites (2008–2024)
Source: Front Immunol. 2025 Jan 24;16:1498453. doi: 10.3389/fimmu.2025.1498453 (PMC11802541; doi:10.3389/fimmu.2025.1498453)
Supplement: Supplementary file 1 [file DataSheet1.pdf]

Supplementary Material

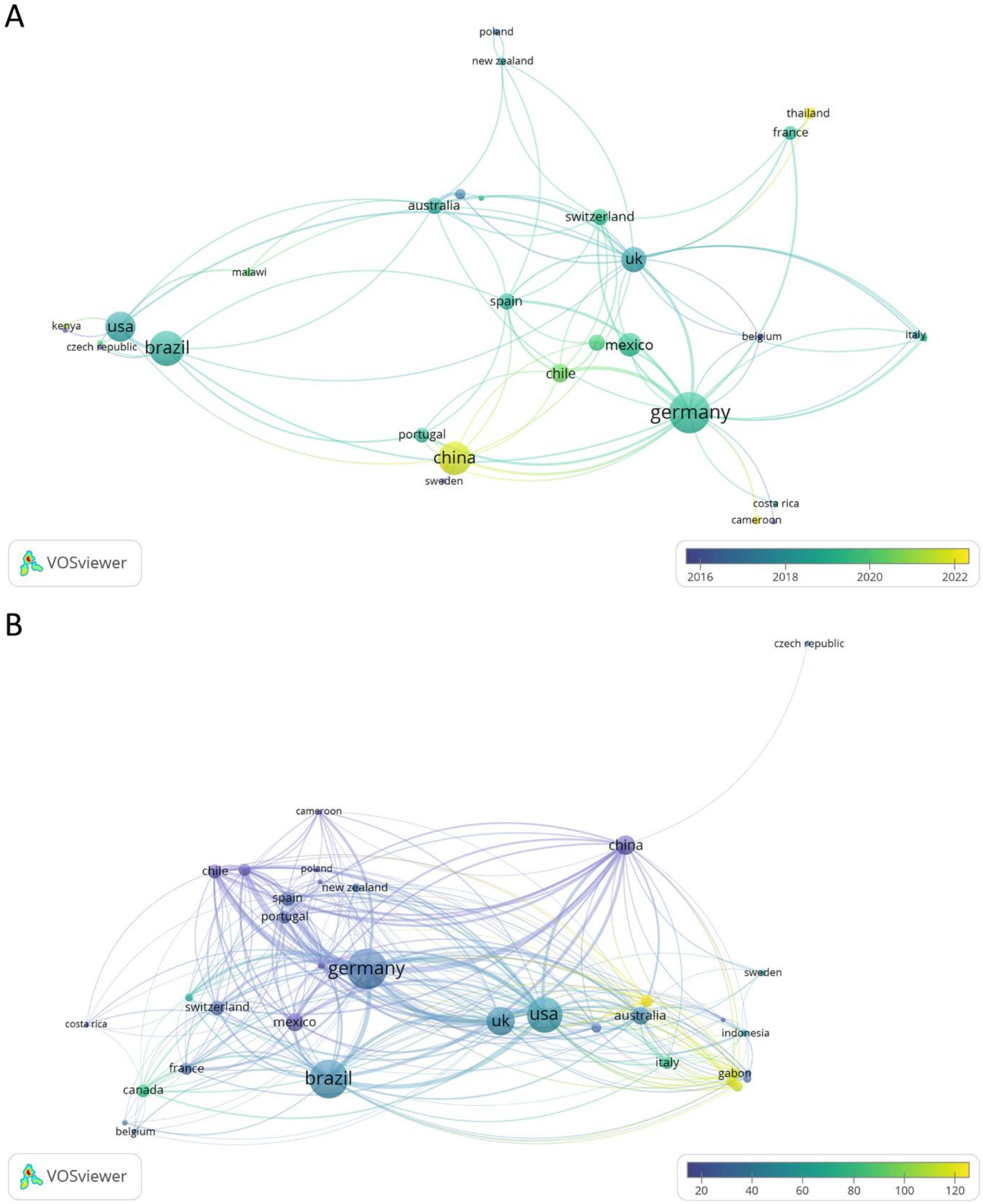

Supplementary Figure 1 – Country contributions to NETs and parasite studies. (**A**, **B**) Network map of countries involved in the research subject. The color of the nodes represents either the average publication year (**A**) or the average number of citations per country (**B**). Node size indicates the number of documents (**A**) or the total number of citations (**B**) produced by each country. Line thickness corresponds to the strength of inter-country collaboration. The analysis in (**B**) includes countries with ten or more citations.

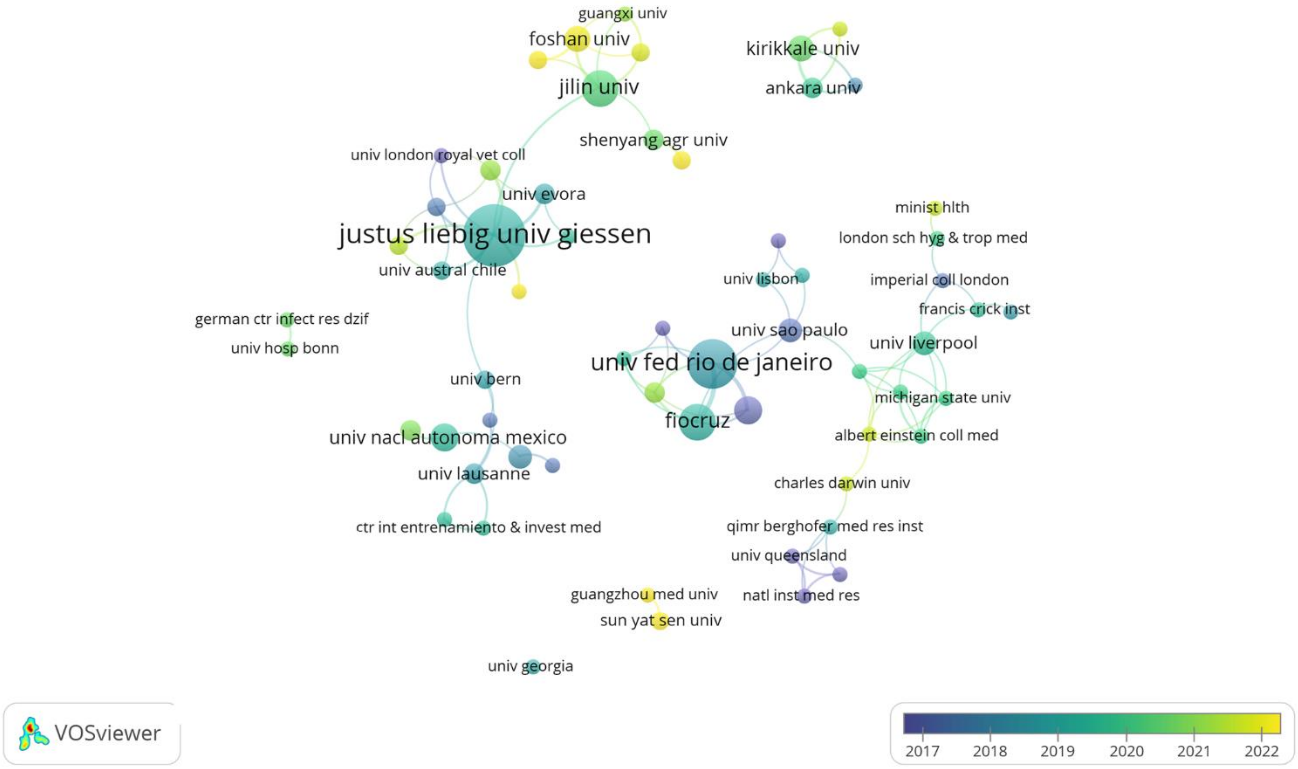

Supplementary Figure 2 – Contribution of research institutions to NETs and parasite studies. Collaborative network map of institutions that have published at least two articles in this area. The color of the nodes represents the average publication year, while the size of the nodes indicates the number of documents produced by each country. The thickness of the lines corresponds to the strength of inter-country collaboration.

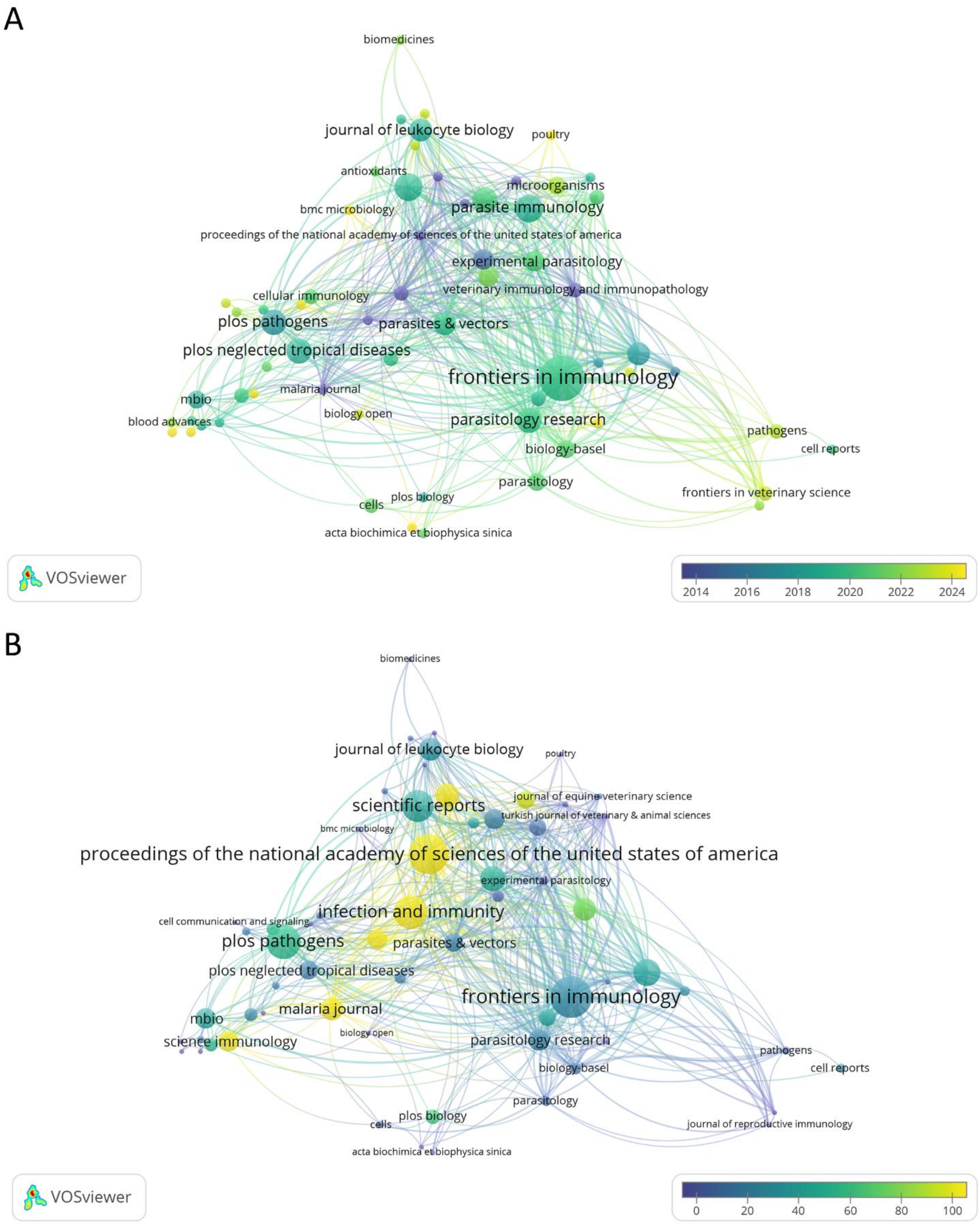

Supplementary Figure 3 – Journal contributions to NETs and parasite studies. (**A**, **B**) Network map of journals involved in the research subject. The color of the nodes represents either the average publication year (**A**) or the average number of citations per journal (**B**), while node size indicates the number of documents (**A**) or the total number of citations (**B**) by each journal. The thickness of the lines corresponds to the strength of citation relationships between journals.

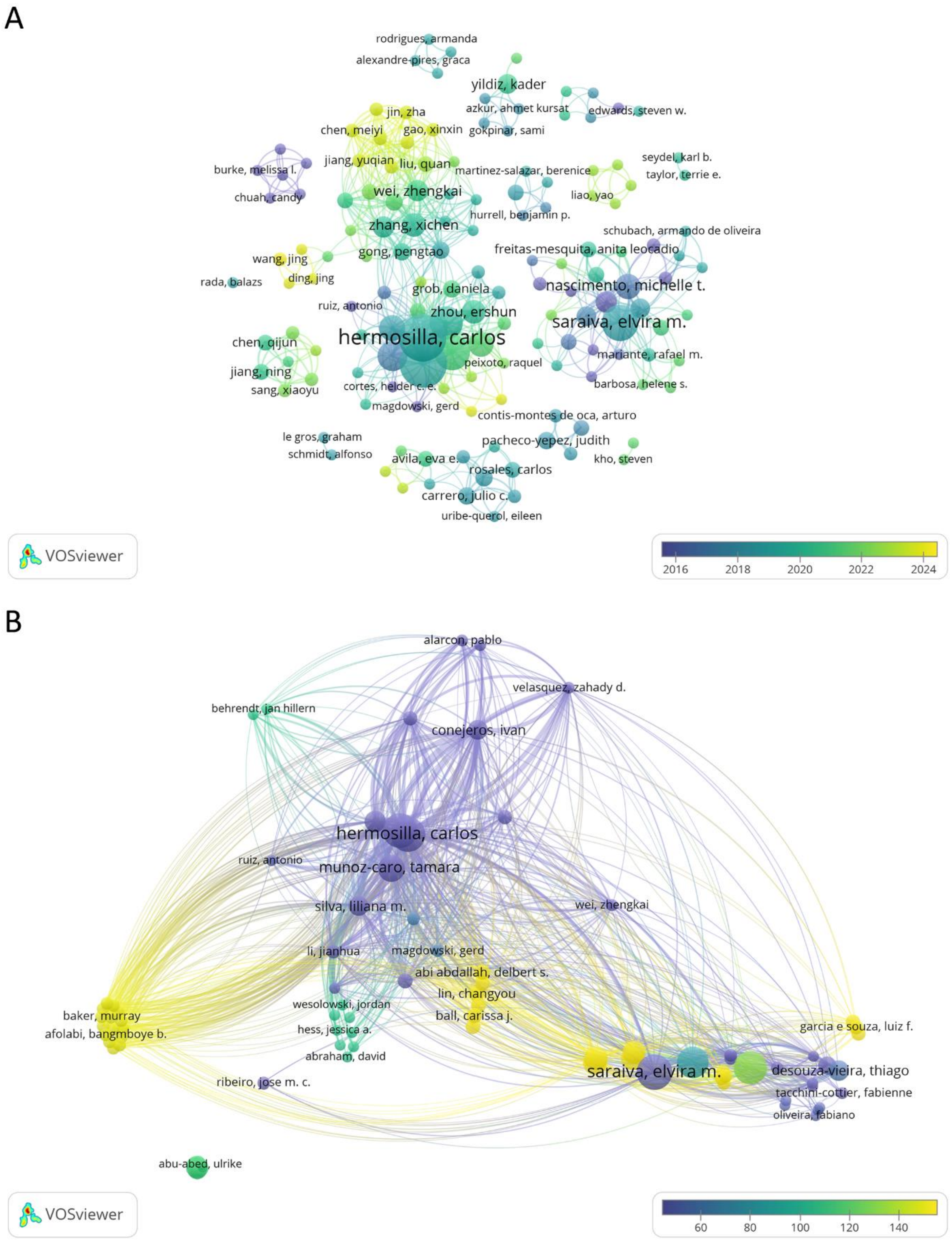

Supplementary Figure 4 – Author contributions to NETs and parasite studies. (**A**, **B**) Collaborative network map of authors involved in the research subject. Node colors represent either the average publication year (**A**) or the average number of citations per author (**B**), while node size indicates the number of documents (**A**) or the total number of citations (**B**) for each author. Lines thickness reflects the strength of collaboration (**A**) or citation relationships (**B**) between authors. The analysis includes authors with at least two published documents (**A**) or with 100 or more citations (**B**).

Top 25 References with the Strongest Citation Bursts

References	Year	Strength	Begin	End	2008 - 2024
Fuchs TA, 2007, J CELL BIOL, V176, P231, DOI 10.1083/jcb.200606027, DOI	2007	3.58	**2008**	2012	
Guimaraes-Costa AB, 2009, P NATL ACAD SCI USA, V106, P6748, DOI 10.1073/pnas.0900226106, DOI	2009	5.24	**2010**	2014	
Gabriel C, 2010, J IMMUNOL, V185, P4319, DOI 10.4049/jimmunol.1000893, DOI	2010	6.74	**2012**	2015	
Abdallah DSA, 2012, INFECT IMMUN, V80, P768, DOI 10.1128/IAI.05730-11, DOI	2012	6.55	**2014**	2017	
Guimaraes-Costa Anderson B, 2012, J PARASITOL RES, V2012, P929743, DOI 10.1155/2012/929743, DOI	2012	5.01	**2014**	2016	
Behrendt JH, 2010, VET IMMUNOL IMMUNOP, V133, P1, DOI 10.1016/j.vetimm.2009.06.012, DOI	2010	4.32	**2014**	2015	
Brinkmann V, 2012, J CELL BIOL, V198, P773, DOI 10.1083/jcb.201203170, DOI	2012	5.08	**2015**	2017	
Silva LMR, 2014, PARASITOL RES, V113, P2797, DOI 10.1007/s00436-014-3939-0, DOI	2014	4.43	**2015**	2019	
Hermosilla C, 2014, PARASITOLOGY, V141, P1489, DOI 10.1017/S0031182014000316, DOI	2014	4.19	**2015**	2018	
Bonne-Année S, 2014, MICROBES INFECT, V16, P502, DOI 10.1016/j.micinf.2014.02.012, DOI	2014	3.84	**2015**	2018	
Hahn S, 2013, SEMIN IMMUNOPATHOL, V35, P439, DOI 10.1007/s00281-013-0380-x, DOI	2013	3.79	**2015**	2017	
Douda DN, 2015, P NATL ACAD SCI USA, V112, P2817, DOI 10.1073/pnas.1414055112, DOI	2015	3.93	**2016**	2019	
Muñoz-Caro T, 2015, DEV COMP IMMUNOL, V52, P245, DOI 10.1016/j.dci.2015.05.007, DOI	2015	3.93	**2016**	2019	
Reichel M, 2015, DEV COMP IMMUNOL, V50, P106, DOI 10.1016/j.dci.2015.02.002, DOI	2015	3.82	**2016**	2020	
Yildiz K, 2017, VET IMMUNOL IMMUNOP, V189, P1, DOI 10.1016/j.vetimm.2017.05.005, DOI	2017	3.89	**2018**	2020	
Villagra-Blanco R, 2017, FRONT IMMUNOL, V8, P0, DOI 10.3389/fimmu.2017.00606, DOI	2017	5.98	**2019**	2022	
Wei ZK, 2016, FRONT IMMUNOL, V7, P0, DOI 10.3389/fimmu.2016.00436, DOI	2016	4.38	**2019**	2021	
Muñoz-Caro T, 2018, FRONT IMMUNOL, V9, P0, DOI 10.3389/fimmu.2018.00968, DOI	2018	6.3	**2020**	2024	
Knackstedt SL, 2019, SCI IMMUNOL, V4, P0, DOI 10.1126/sciimmunol.aaw0336, DOI	2019	4.05	**2020**	2024	
Zhou ES, 2019, FRONT IMMUNOL, V10, P0, DOI 10.3389/fimmu.2019.01131, DOI	2019	3.67	**2020**	2024	
Imlau M, 2020, DEV COMP IMMUNOL, V103, P0, DOI 10.1016/j.dci.2019.103527, DOI	2020	3.67	**2020**	2024	
Silva LMR, 2016, MEDIAT INFLAMM, V2016, P0, DOI 10.1155/2016/5898074, DOI	2016	3.63	**2020**	2021	
Conejeros I, 2019, BIOLOGY-BASEL, V8, P0, DOI 10.3390/biology8040078, DOI	2019	5.12	**2021**	2024	
Grob D, 2020, FRONT IMMUNOL, V11, P0, DOI 10.3389/fimmu.2020.559561, DOI	2020	5.12	**2021**	2024	
Bouchery T, 2020, CELL HOST MICROBE, V27, P277, DOI 10.1016/j.chom.2020.01.011, DOI	2020	3.71	**2021**	2024	

Supplementary Figure 5 – Top 25 co-cited references with the strongest citation burst. Articles indicated by a red arrow are among the 159 papers included in our analysis.

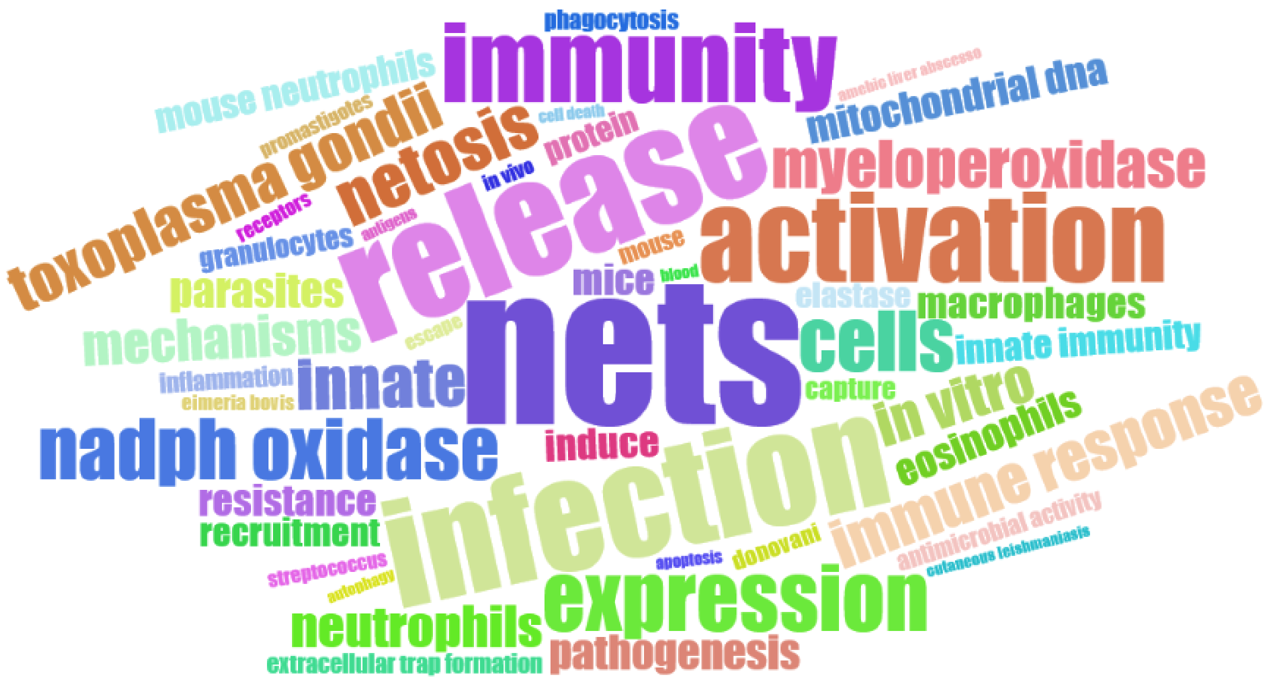

Supplementary Figure 6 – A word cloud of the 50 most frequently used research keywords in the field of NETs and parasites.

Supplementary Table 1 – Top 10 countries contributing to the research area ranked by the number of citations followed by the number of documents published.

Rank	Country	Articles	Citations	Citations/Articles
1	Germany	45	1,495	33,22
2	Brazil	32	1,342	41,94
3	USA	24	1,147	47,79
4	UK	17	713	41,94
5	China	32	337	10,53
6	Mexico	15	293	19,53
7	Australia	7	280	40,00
8	Spain	7	198	28,29
9	Switzerland	7	197	28,14
10	Chile	9	185	20,56

Supplementary Table 2 – Top 10 most productive institutions contributing to the research field with two or more documents, ranked by the number of citations followed by the number of articles.

Rank	Institution	Country	Articles	Citations
1	Federal University of Rio de Janeiro	Brazil	22	1105
2	Justus Liebig University Giessen	Germany	35	995
3	Fiocruz	Brazil	12	636
4	National Institute of Allergy and Infectious Diseases	USA	7	412
5	Jilin University	China	12	235
6	University of Liverpool	UK	5	174
7	Francis Crick Institute	UK	2	164
8	National Autonomous University of Mexico	Mexico	7	157
9	Imperial College London	UK	2	146
10	University of São Paulo	Brazil	5	142

Supplementary Table 3 – Top 10 journals publishing in the field ranked by the number of citations.

Rank	Journal	Articles	Citations	JIF (2024)	JCR
1	*Frontiers in Immunology*	19	477	5.7	Q1
2	*Proceedings of the National Academy of Sciences of the United States of America*	1	438	9.4	Q1
3	*PLoS Pathogens*	6	318	5.5	Q1
4	*Infection and Immunity*	2	300	2.9	Q2
5	*Scientific Reports*	7	284	3.8	Q1
6	*Developmental and Comparative Immunology*	5	201	2.7	Q1
7	*PLoS One*	4	181	2.9	Q1
8	*Journal of Immunology*	1	156	3.6	Q2
9	*Veterinary Immunology and Immunopathology*	2	147	1.4	Q2
10	*Journal of Leukocyte Biology*	5	146	3.6	Q2

Supplementary Table 4 – Top 10 researchers in the field with two or more documents, ranked by the number of citations.

Rank	Author	Articles	Citations	*h*-Index*
1	Saraiva EM	21	1,070	12
2	Hermosilla CR	35	995	19
3	Taubert A	35	995	19
4	Guimarães-Costa AB	7	898	6
5	Nascimento MTC	10	850	8
6	Muñoz-Caro T	14	609	12
7	Conceição-Silva F	3	468	2
8	Morgado FN	3	468	2
9	Gaertner U	17	387	11
10	DeSouza-Vieira TS	6	364	5

*The h-index reported here is calculated based solely on the papers included in this analysis, reflecting the authors' impact within the scope of the study.

Supplementary Table 5 – Top 10 co-cited references in the research field of NETs and parasites ranked by the number of citations.

Rank	Co-cited reference	Citations
1	Brinkmann V, 2004, *Science*, v303, p1532, doi 10.1126/science.1092385	116
2	Guimaraes-Costa AB, 2009, *Proc Natl Acad Sci USA*, v106, p6748, doi 10.1073/pnas.0900226106	75
3	Fuchs TA, 2007, *J Cell Biol*, v176, p231, doi 10.1083/jcb.200606027	67
4	Abi Abdallah DS, 2012, *Infect Immun*, v80, p768, doi 10.1128/iai.05730-11	59
5	Papayannopoulos V, 2010, *J Cell Biol*, v191, p677, doi 10.1083/jcb.201006052	47
6	Behrendt JH, 2010, *Vet Immunol Immunophatol*, v133, p1, doi 10.1016/j.vetimm.2009.06.012	45
7	Gabriel C, 2010, *J Immunol*, v185, p4319, doi 10.4049/jimmunol.1000893	41
8	Baker VS, 2008, *Malaria J*, v7, 41, doi 10.1186/1475-2875-7-41	40
9	Urban CF, 2006, *Cell Microbiol*, v8, p668, doi 10.1111/j.1462-5822.2005.00659.x	39
10	Urban CF, 2009, *Plos Pathog*, v5, e1000639, doi 10.1371/journal.ppat.1000639	36

Supplementary Table 6 – Top 20 most frequently cited keywords in the research field ranked by the number of occurrences.

Rank	Keyword	Occurrences	Rank	Keyword	Occurrences
1	nets	98	11	expression	20
2	neutrophils	52	12	in vitro	20
3	release	35	13	nadph oxidase	19
4	infection	33	14	myeloperoxidase	18
5	netosis	32	15	innate	16
6	toxoplasma gondii	29	16	immune response	16
7	activation	27	17	mechanisms	13
8	immunity	25	18	mice	12
9	innate immunity	25	19	mouse neutrophils	12
10	cells	20	20	reactive oxygen species	12
